# Artificial Intelligence Models for Mortality and Outcome Prediction in Intensive Care Unit Sepsis: A Systematic Review

**DOI:** 10.3390/jpm16070346

**Published:** 2026-06-25

**Authors:** Giuseppe Mazza, Giuseppe Neri, Helenia Mastrangelo, Alessandro Russo, Isabella Aquila, Matteo Antonio Sacco, Jessica Ielapi, Corrado Pelaia, Mario Cannataro, Chiara Lupia, Francesca Serapide, Federico Longhini, Vincenzo Bosco, Zaninni Caroleo, Andrea Bruni, Eugenio Garofalo, the SEPSIS-UMG Collaborative Group

**Affiliations:** 1 Department of Medical and Surgical Sciences, University “Magna Graecia” of Catanzaro, 88100 Catanzaro, Italy; giuseppe.mazza@unicz.it (G.M.); giuseppeneri91@gmail.com (G.N.); a.russo@unicz.it (A.R.); isabella.aquila@unicz.it (I.A.); matteosacco@unicz.it (M.A.S.); jessica.ielapi22@gmail.com (J.I.); pelaia.corrado@unicz.it (C.P.); cannataro@unicz.it (M.C.); f.serapide@unicz.it (F.S.); flonghini@unicz.it (F.L.); vincenzo.bosco@unicz.it (V.B.); 2 Department of Health Sciences, University “Magna Graecia” of Catanzaro, 88100 Catanzaro, Italy; heleniamastrangelo@gmail.com; 3 ASP Catanzaro, 88100 Catanzaro, Italy; chiaralupia1996@gmail.com; 4 Department of Pharmacy, Health and Nutritional Sciences, University of Calabria, 87036 Rende, Italy; caroleozaninni@gmail.com (Z.C.); andrea.bruni@unical.it (A.B.)

**Keywords:** sepsis, septic shock, intensive care unit, artificial intelligence, machine learning, mortality prediction, PROBAST, TRIPOD

## Abstract

**Background/Objectives:** Artificial intelligence (AI), machine-learning (ML), and deep-learning (DL) models are increasingly used for prognostic prediction in intensive care unit (ICU) sepsis, but their clinical readiness remains uncertain. This systematic review aimed to evaluate AI-, ML-, and DL-based models for mortality and clinically relevant outcome prediction in adult ICU patients with sepsis or septic shock. **Methods**: PubMed/MEDLINE, Scopus, and the Cochrane Library were searched up to April 2026. Eligible studies included adult ICU sepsis or septic shock cohorts evaluating AI/ML/DL-based prognostic models. Screening, full-text assessment, and data extraction were performed independently by two reviewers. Outcomes, model families, validation strategies, discrimination, calibration, clinical utility, explainability, comparative performance versus conventional severity scores, risk of bias, and reporting completeness were synthesized. Risk of bias was assessed using PROBAST domains supplemented by PROBAST + AI considerations, and reporting completeness was evaluated according to TRIPOD/TRIPOD + AI domains. **Results**: Seventy-five studies were included, comprising 50 PubMed-derived and 25 additional Scopus-derived studies. AUROC or C-statistic was extractable in 64 studies, external validation was reported in 27, prospective evaluation in three, calibration in 38, decision-curve analysis or clinical utility assessment in 37, and explainability in 64. Across 17 directly extractable within-study comparisons from nine studies, AI/ML models usually, but not uniformly, achieved higher discrimination than conventional severity scores, with a median paired ΔAUROC of +0.108 (IQR, +0.082 to +0.148; range, −0.013 to +0.203). Externally validated fixed-horizon models showed clinically relevant but heterogeneous discrimination across sepsis phenotypes, with stronger evidence in selected sepsis-induced coagulopathy cohorts and more variable transportability in respiratory and liver-injury subgroups. However, 45 studies were judged at high risk of bias, mainly because of limitations in the analysis domain. **Conclusions:** AI/ML models for adult ICU sepsis show a recurrent signal of prognostic discrimination and often perform comparably to or better than conventional severity scores in directly extractable within-study comparisons; however, this signal should be interpreted cautiously given clinical and methodological heterogeneity, limited prospective validation, incomplete calibration, and frequent high risk of bias. The strongest evidence comes from externally validated, phenotype-specific models, although routine clinical implementation remains limited by heterogeneous endpoints, incomplete calibration, insufficient prospective validation, and scarce workflow-level evaluation. Future studies should shift from retrospective AUROC optimization toward calibrated, externally validated, clinically actionable, and workflow-integrated decision-support tools tested in prospective ICU settings.

## 1. Introduction

Sepsis remains one of the most complex and high-risk syndromes encountered in adult intensive care units (ICUs). According to the Sepsis-3 consensus definition, sepsis is life-threatening organ dysfunction caused by a dysregulated host response to infection [1]. In critically ill patients, this syndrome may progress rapidly to septic shock, multiple-organ dysfunction, prolonged organ support, long-term morbidity, and death. Despite major advances in early recognition, antimicrobial therapy, source control, hemodynamic resuscitation, and organ-support strategies, bedside prognostication remains challenging because patients with apparently similar clinical presentations may follow markedly different trajectories [2].

The most recent Surviving Sepsis Campaign guidelines for adult sepsis and septic shock emphasize timely diagnosis; structured clinical decision-making; early treatment; source control; hemodynamic optimization; and continuity of care across the prehospital, hospital, ICU and post-discharge phases [2]. However, these recommendations also highlight a central challenge in sepsis care: treatment decisions must often be made before the individual patient trajectory is fully apparent. Mortality risk is influenced by baseline vulnerability, comorbidities, infection source, microbiology, immune-inflammatory response, organ dysfunction pattern, timing and adequacy of interventions, and exposure to ICU therapies. This complexity explains why reliable, individualized and dynamically updated outcome prediction remains a major unmet need in critical care.

Risk stratification in sepsis has traditionally relied on clinical judgement and severity scores such as the Sequential Organ Failure Assessment (SOFA), quick SOFA (qSOFA), Acute Physiology and Chronic Health Evaluation (APACHE), Simplified Acute Physiology Score (SAPS), Logistic Organ Dysfunction System (LODS), Oxford Acute Severity of Illness Score (OASIS), and related organ dysfunction or acuity indices [3,4,5,6,7,8]. These instruments remain useful for describing illness severity, standardizing communication, prognostic stratification, and supporting population-level comparisons [3,4,5,6,7,8]. Nevertheless, they were not designed to capture the full complexity of individualized prognosis across heterogeneous ICU sepsis phenotypes. In particular, static or single-time-point scores may incompletely reflect nonlinear interactions, high-dimensional covariate structures, time-varying physiological patterns, treatment-response trajectories, and evolving organ dysfunction.

The centrality of organ dysfunction as both a prognostic marker and an outcome endpoint is also reflected in contemporary interventional sepsis research, where organ dysfunction and clinical outcomes are commonly used to capture disease trajectory and treatment response [9,10]. Although this line of investigation was not focused on artificial intelligence, it illustrates a broader principle relevant to prognostic modeling: sepsis outcome prediction is clinically meaningful only when it captures the dynamic relationship between host response, organ failure, and treatment course.

Artificial intelligence (AI), machine-learning (ML), and deep-learning (DL) approaches have increasingly been proposed as tools to improve prognostic modeling in critical care. These methods can integrate high-dimensional electronic health record data and, in selected studies, longitudinal physiological signals, laboratory trajectories, treatment variables, organ-support parameters, imaging, omics, or multimodal features. In principle, they may identify nonlinear relationships and temporal patterns that are difficult to model using conventional regression-based tools or static severity scores. The availability of large ICU databases, including MIMIC-III, MIMIC-IV, and eICU, has accelerated the development and validation of AI-based prediction models in adult critical care [11,12,13].

However, discrimination alone does not establish clinical usefulness. A prognostic model intended for ICU decision support should also demonstrate calibration, transportability, reproducibility, clinical utility, interpretability, and feasibility of integration into real-world workflows. This distinction is particularly important in sepsis, where retrospective database-derived performance may be inflated by local case-mix, measurement frequency, missingness patterns, coding practices, treatment protocols, temporal leakage, or overfitting. Therefore, systematic evaluation of AI-based prognostic models in sepsis should not focus only on reported AUROC values, but also on validation strategy, calibration, clinical utility, reporting completeness, and risk of bias.

Methodological standards for prediction model research have evolved to address these concerns. PRISMA 2020 provides the reporting framework for systematic reviews [14], while PROBAST/PROBAST + AI and TRIPOD/TRIPOD + AI support structured assessment of risk of bias, applicability, and reporting completeness in clinical prediction models, including AI-based models [15,16,17,18]. Applying these frameworks to ICU sepsis AI models is essential to distinguish promising algorithmic performance from evidence that is sufficiently validated, transparently reported, and clinically transferable.

In adult ICU sepsis, AI/ML/DL models have been applied to a broad range of prognostic endpoints, including ICU mortality, in-hospital mortality, 28-day and 30-day mortality, long-term mortality, organ dysfunction, treatment response, readmission, length of stay, and composite clinical outcomes, as detailed in Supplementary Table S3. Many studies have reported promising discrimination, and several have suggested that AI/ML models may outperform conventional severity scores. However, the apparent expansion of AI-based prognostic modeling has not automatically translated into clinical implementation. Whether these models are adequately validated, transparently reported, clinically useful, and ready for bedside integration remains uncertain.

Accordingly, this systematic review aimed to identify, synthesize and critically appraise studies evaluating AI-, ML-, or DL-based models for mortality and clinically relevant outcome prediction in adult ICU patients with sepsis or septic shock. Specifically, we summarized study populations, data sources, outcome definitions, prediction horizons, model families, validation strategies, performance metrics, calibration, clinical utility, explainability, and comparative performance versus conventional severity scores, while also appraising risk of bias according to PROBAST/PROBAST + AI principles and reporting completeness according to TRIPOD/TRIPOD + AI domains.

## 2. Materials and Methods

### 2.1. Study Design and Reporting Framework

This systematic review was designed to identify, characterize, and critically appraise studies evaluating artificial intelligence (AI), machine-learning (ML), or deep-learning (DL) models for mortality and clinically relevant outcome prediction in adult intensive care unit (ICU) patients with sepsis or septic shock. The review was conducted and reported in accordance with the PRISMA 2020 statement [14]. The completed PRISMA 2020 checklist is provided in Supplementary Table S8. Risk of bias and applicability were assessed using PROBAST domains, supplemented by PROBAST + AI considerations for AI-based prediction models [15,16]. Reporting completeness was evaluated according to TRIPOD and TRIPOD + AI domains [17,18]. The review protocol was not prospectively registered. This represents a methodological limitation. The omission was due to the fact that the project was initially conceived as a structured evidence synthesis of AI-based prognostic models in ICU sepsis and was subsequently expanded into a full PRISMA 2020 systematic review after database updating, full-text reassessment, and refinement of the adult ICU focus. To reduce the risk of selective reporting, eligibility criteria, complete search strategies, screening flow, full-text exclusion reasons, data-extraction fields, risk-of-bias domains, reporting-completeness items, and synthesis rules are reported in detail in the Section 2 and Supplementary Materials.

### 2.2. Research Question and PICO Framework

The review question was structured according to the PICO framework to define eligibility criteria and guide synthesis. The population of interest included adult patients admitted to ICUs or critical-care settings with sepsis or septic shock, as defined by the original study authors. Studies based on ICU-derived databases were considered eligible when the source population was adult critical-care patients and the target cohort consisted of patients with sepsis, septic shock, or a clearly defined adult ICU sepsis subgroup. Eligible subgroups included, but were not limited to, septic shock; sepsis-associated acute kidney injury; sepsis-associated liver injury; sepsis-associated delirium or encephalopathy; sepsis-induced coagulopathy; sepsis-related acute respiratory failure or ARDS; cancer-associated sepsis; diabetes-associated sepsis; postoperative sepsis; urosepsis; and ICU readmission after sepsis.

The index model was any AI-, ML-, or DL-based prognostic model developed, validated, updated, or evaluated for mortality or clinically relevant outcome prediction. Eligible approaches included supervised machine-learning models, tree-based ensembles, boosting algorithms, random forests, support vector machines, neural networks, deep-learning architectures, time-series models, survival machine-learning models, multimodal models, imaging- or omics-based models, and large-language-model- or guideline-integrated prediction approaches. Studies evaluating conventional regression models alone were not eligible unless these models were used as benchmarks, comparators, or components of a broader AI/ML modeling framework.

Comparators included conventional statistical models; clinician-derived benchmarks; conventional severity scores such as SOFA, qSOFA, APACHE, SAPS, OASIS, or LODS; alternative AI/ML algorithms; or no comparator, depending on the original study design [3,4,5,6,7,8]. Outcomes included mortality and clinically relevant prognostic endpoints; including ICU mortality; hospital or in-hospital mortality; 7-day, 28-day, 30-day, 90-day, or longer-term mortality; organ dysfunction; clinical deterioration; readmission; length of stay; treatment response; severity trajectories; and composite clinical outcomes. Diagnostic-only studies, models intended solely to identify sepsis onset, and models without a prognostic mortality or clinical outcome endpoint were outside the scope of this review.

### 2.3. Information Sources and Search Strategy

A systematic literature search was performed in PubMed/MEDLINE and Scopus. The search covered studies published up to April 2026. The PubMed/MEDLINE search combined terms related to sepsis, septic shock, intensive care, artificial intelligence, machine learning, deep learning, prediction models, prognosis, mortality, and clinical outcomes. Filters were applied for human studies, English language, article type, and publication within the last 10 years. A complementary Scopus search was performed to identify additional studies not retrieved in the PubMed-derived corpus.

The Cochrane Library was also searched to verify whether additional eligible records were available. No additional eligible AI/ML-based prognostic prediction studies in adult ICU sepsis were identified through the Cochrane search, and the Cochrane search did not modify the final study set. The complete reproducible database-specific search strategies, including the full PubMed/MEDLINE, Scopus, and Cochrane Library search strings; applied filters; search dates; and record counts, are reported in Supplementary Table S1. This Supplementary table is intended to allow full replication of the search process and should be considered part of the PRISMA 2020 reporting documentation.

### 2.4. Eligibility Criteria

Studies were eligible if they met all of the following criteria: original research; adult ICU or critical-care population; patients with sepsis or septic shock, or a clearly defined adult ICU sepsis subgroup; development, validation, updating, or evaluation of an AI-, ML-, or DL-based prognostic model; and prediction of mortality or another clinically relevant outcome. Studies were considered eligible irrespective of whether the model was developed using electronic health record data, laboratory variables, physiological time-series, imaging, omics, multimodal data, or publicly available ICU databases.

Studies were excluded if they were reviews, editorials, commentaries, letters without original data, conference abstracts without sufficient methodological detail, non-human studies, pediatric or neonatal-only studies, non-ICU populations, diagnostic-only studies without prognostic outcome prediction, studies not focused on sepsis or septic shock, or studies that did not evaluate an AI/ML/DL-based prognostic model. Studies evaluating mixed ICU populations were included only when adult sepsis or septic shock represented the primary target population, or when sepsis-specific adult ICU results were clearly extractable from the article or supplementary material. Studies in which sepsis patients were embedded within broader ICU cohorts without separable sepsis-specific results were excluded. Similarly, mixed adult–pediatric studies were retained only if adult ICU sepsis data were reported separately; otherwise, they were excluded. Borderline cases were resolved by full-text reassessment and consensus discussion between reviewers, with priority given to preserving a clinically homogeneous adult ICU sepsis population. Mortality-only studies were eligible when the population, setting, and model type matched the review question; diagnostic-only sepsis detection models were excluded unless they also evaluated a prognostic mortality or clinical outcome endpoint.

### 2.5. Study Selection

Records identified through PubMed/MEDLINE, Scopus, and the Cochrane Library were screened according to the predefined eligibility criteria. Two independent reviewers screened titles and abstracts for eligibility. Full-text articles were assessed independently, and any discrepancies were resolved by consensus. Duplicate records, pediatric or neonatal studies, non-ICU studies, diagnostic-only models, non-sepsis populations, and studies without AI/ML-based prognostic outcome prediction were excluded. When eligibility remained uncertain, the full text was re-assessed, and the final decision was reached through discussion between reviewers.

**Figure 1:**
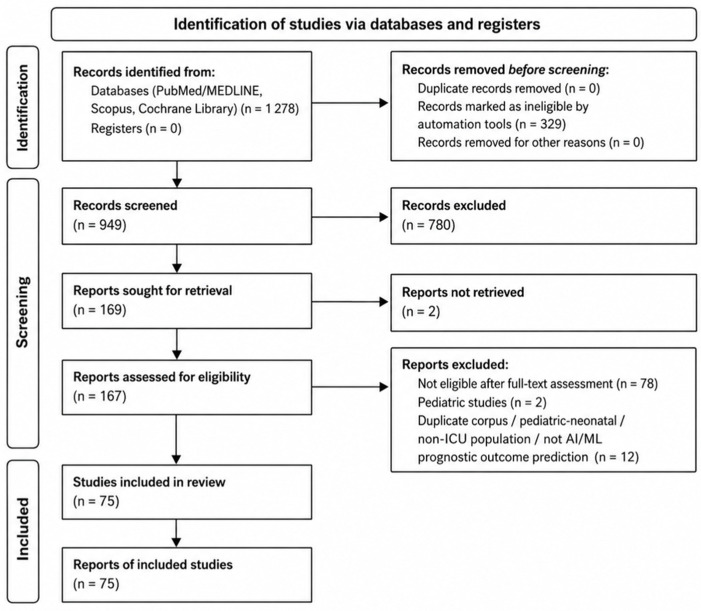
PRISMA 2020 flow diagram of study selection. AI/ML, artificial intelligence/machine learning; ICU, intensive care unit.

Duplicate reports were handled at both the database and full-text stages. Studies already included in the PubMed-derived corpus were not counted again when retrieved through Scopus. When multiple versions, duplicate files, or overlapping reports were identified, the most complete full-text report was retained. Our study selection process is summarized in the PRISMA flow diagram (Figure 1). Reasons for full-text exclusion are reported in Supplementary Table S2. The PRISMA flow diagram reports the number of records identified, screened, excluded, assessed in full text, and included in the final synthesis. Full-text exclusion categories are additionally summarized in Supplementary Table S2.

### 2.6. Data Extraction

Data extraction was performed independently by two reviewers using a structured extraction form, and disagreements were resolved through discussion. For each included study, the following information was collected: first author; year of publication; country or setting; study design; data source; sample size; sepsis definition; population characteristics; ICU setting; prediction target; outcome definition; prediction time horizon; time zero; feature window; data type; model family; best-performing or proposed model; comparator models or conventional severity scores; validation strategy; performance metrics; calibration; decision-curve analysis or clinical utility assessment; explainability methods; model availability; code availability; and implementation features.

Performance metrics included AUROC or C-statistic; AUPRC, when reported; accuracy; sensitivity; specificity; F1 score; precision; recall; calibration metrics; Brier score; and decision-curve or net-benefit analyses. When multiple models were reported, the model identified by the original authors as the best-performing, final, or clinically proposed model was preferentially extracted. When multiple validation cohorts were reported, internal and external validation results were recorded separately whenever possible. When performance estimates were reported separately for development, internal validation, temporal validation, and external validation cohorts, external validation was prioritized for clinical interpretation. The complete extraction table for all included studies is provided in Supplementary Table S3.

### 2.7. Data Items and Effect Measures

The primary effect measure of model discrimination was AUROC or C-statistic, depending on the terminology used in the original study. AUPRC was extracted when reported, particularly because class imbalance may affect interpretation in mortality or deterioration prediction. Calibration was extracted as reported by the original authors, including calibration plots, calibration slope, calibration in the large, Brier score, Hosmer–Lemeshow testing, or qualitative statements on calibration. Clinical utility was extracted when studies reported decision-curve analysis, net benefit, threshold analysis, risk stratification, online calculators, nomograms, web tools, or implementation-oriented outputs.

For studies comparing AI/ML models with conventional scores, the main comparative effect measure was the paired difference in discrimination, calculated descriptively as ΔAUROC. This was defined as the AUROC of the AI/ML model minus the AUROC of the best-performing conventional comparator within the same study, outcome, and validation cohort. Because most studies did not report paired uncertainty for ΔAUROC, this measure was used for descriptive synthesis only. For the final ΔAUROC calculation, only comparisons with both AI/ML and comparator-score AUROC/C-statistic values directly extractable from the full text or uploaded Supplementary Materials were retained; comparisons available only graphically or without exact numeric values were excluded from the calculation.

### 2.8. Outcome Classification

Outcomes were grouped into clinically meaningful domains before synthesis. Mortality outcomes were classified as ICU mortality, hospital or in-hospital mortality, early or short-term mortality, 28-day mortality, 30-day mortality, long-term or survival-type mortality, mortality with unspecified or variable time horizon, and composite or multi-outcome mortality prediction. Other clinically relevant outcomes included organ dysfunction, sepsis-associated complications, clinical deterioration, readmission, length of stay, treatment response, severity trajectories, and composite prognostic outcomes.

When studies reported multiple outcomes, each outcome was mapped separately when relevant. For tabular summaries, each study was classified according to the primary or most clinically emphasized outcome in order to avoid double-counting in descriptive statistics. Secondary outcomes and additional model outputs were retained in the complete extraction table and considered in the narrative synthesis.

### 2.9. Model Classification

Models were grouped according to their primary modeling approach. Categories included boosting methods such as XGBoost, LightGBM, CatBoost, gradient boosting, and gradient-boosting decision tree models; random forest and other ensemble methods; support vector machine and other classical machine-learning approaches; neural networks and deep-learning models, including artificial neural networks, recurrent neural networks, long short-term memory networks, Transformer-based models, and graph neural networks; survival machine-learning models; multimodal, imaging, or omics-based models; large-language-model- or guideline-integrated approaches; and traditional statistical or hybrid models when used as part of AI/ML pipelines.

When several algorithms were tested in the same study, classification was based on the final, best-performing or clinically emphasized model as reported by the original authors. Because many studies evaluated multiple algorithms, model-family categories were used for structured synthesis rather than as mutually exclusive descriptions of all algorithms tested.

### 2.10. Data Synthesis of AI/ML–Conventional Score Comparisons

Because several studies directly compared AI/ML models with conventional severity scores, a dedicated comparative synthesis was performed. Eligible comparisons required the same study or cohort, and the same outcome and reported AUROC/C-statistic for both an AI/ML model and a conventional score or score-derived comparator. Conventional comparators included SOFA, qSOFA, APACHE II/III, SAPS II, APS-III, LODS, OASIS, and SOFA-derived trajectories [3,4,5,6,7,8]. When multiple conventional scores were available in the same cohort, the best-performing score was selected to provide a conservative estimate of the difference between AI/ML and conventional scoring.

The paired difference in discrimination was summarized descriptively as ΔAUROC, calculated as the AUROC of the AI/ML model minus the AUROC of the conventional comparator. Importantly, AUROC values from AI/ML models and conventional scores were not pooled as independent estimates across different studies; only within-study paired comparisons were considered for descriptive ΔAUROC calculation. Formal meta-analysis of ΔAUROC was not performed because most studies did not report paired uncertainty estimates, DeLong confidence intervals for the AUROC difference, covariance between AUROC estimates, or sufficient information to reconstruct the standard error of the paired difference.

### 2.11. Risk of Bias and Applicability Assessment

Risk of bias and applicability were assessed using PROBAST domains, supplemented by PROBAST + AI considerations for AI-based prediction models [15,16]. Domains included participants, predictors, outcome, and analysis, with additional AI-specific considerations applied where relevant. Particular attention was paid to patient selection, representativeness, outcome definition, predictor timing, missing-data handling, sample size, events per predictor or model complexity, validation strategy, overfitting, data leakage, hyperparameter tuning, reproducibility, external validation, and clinical applicability.

Each study was assigned an overall judgment of low risk, some concerns, or high risk of bias. Applicability concerns were evaluated in relation to the review question, adult ICU sepsis population, prediction setting, data source, and intended clinical use. Study-level judgments and domain-level summary findings are reported in Supplementary Table S4.

### 2.12. Reporting Completeness Assessment

Reporting completeness was evaluated according to TRIPOD and TRIPOD + AI domains [17,18]. Items assessed included title and abstract, objectives, source of data, eligibility criteria, outcome definition, predictor definition and timing, sample size, missing data, model development, preprocessing, feature selection, hyperparameter tuning, validation, performance metrics, calibration, clinical utility, explainability, model availability, code availability, and limitations.

Reporting was classified as adequate, partial or poor/not reported for each major item group. Study-level reporting assessments and domain-level summary findings are reported in Supplementary Table S5. TRIPOD/TRIPOD + AI was used to evaluate reporting completeness and not to determine methodological validity; its findings were therefore interpreted alongside the PROBAST/PROBAST + AI risk-of-bias assessment.

### 2.13. Data Synthesis

Because substantial heterogeneity was anticipated in population, sepsis definition, outcome horizon, prediction time zero, feature window, data source, model family, validation strategy, and performance reporting, the primary synthesis was planned as a structured qualitative synthesis. Studies were summarized according to outcome domain, model type, validation approach, performance reporting, calibration, clinical utility, explainability, risk of bias, and reporting completeness.

Quantitative synthesis was considered only after data extraction and only for outcome groups with sufficiently comparable populations, prediction horizons, validation settings, and extractable AUROC or C-statistic estimates with corresponding uncertainty. No meta-analysis was performed as the primary synthesis because no outcome cluster met predefined criteria for sufficiently homogeneous population, time horizon, validation strategy and uncertainty reporting. Similarly, meta-analysis of AI/ML versus conventional score differences was not performed because paired ΔAUROC uncertainty, DeLong confidence intervals, or ROC covariance estimates were not consistently reported. Comparative performance was therefore summarized descriptively.

### 2.14. Certainty and Translational Readiness

In addition to risk-of-bias and reporting assessments, studies were narratively evaluated for translational readiness. Elements considered included external validation, prospective evaluation, calibration, decision-curve analysis, explainability, comparison with conventional scores, model availability, code availability, workflow integration, clinical interpretability, and evidence of impact on bedside decision-making. GRADE was not applied because the review focused on prediction model development, validation, and reporting rather than comparative intervention effects.

The feasibility of quantitative synthesis across outcome domains is summarized in Supplementary Table S6.

## 3. Results

### 3.1. Characteristics of the Included Studies

Our study selection process is summarized in Figure 1. The PubMed/MEDLINE search identified 1055 records. After filters for human studies, English language, article type, and publication within the last 10 years were applied, 726 records remained for title and abstract screening. Of these, 132 reports were assessed in full text. In the original PubMed-derived corpus, 54 studies met the initial eligibility criteria. During the adult-only refinement of the review, two pediatric studies were excluded, and two potentially eligible reports were excluded because the full text could not be retrieved. Therefore, 50 PubMed-derived studies were retained for the final adult ICU synthesis.

The complementary Scopus search identified 223 records. After duplicate removal, title and abstract screening, and full-text assessment, 37 full-text reports were evaluated, and 25 additional adult ICU sepsis studies were included. Studies already included in the PubMed-derived corpus, pediatric or neonatal studies, studies not primarily focused on adult ICU sepsis, and reports not evaluating AI/ML-based prognostic outcome prediction were excluded.

The Cochrane Library search did not identify additional eligible adult ICU sepsis AI/ML prognostic prediction studies. Overall, the final qualitative synthesis included 75 studies evaluating AI-, ML-, or DL-based models for mortality or clinically relevant outcome prediction in adult ICU patients with sepsis or septic shock. The complete list of included studies and the study-level extraction data are provided in Supplementary Table S3.

### 3.2. Evidence Profile

Across the 75 included studies, the evidence base was large but unevenly mature. Model development was frequent and discrimination was commonly reported, with AUROC or C-statistic extractable in 64 studies. However, evidence supporting clinical translation was substantially less developed: external validation was reported in 27 studies, prospective evaluation in only 3, calibration in 38, and decision-curve analysis or another clinical utility assessment in 37. Explainability was frequently reported, appearing in 64 studies, but was usually implemented as post hoc feature-importance analysis rather than prospective decision support.

Taken together, these findings indicate that AI/ML modeling in ICU sepsis has progressed substantially in algorithm development and discrimination reporting, but remains less mature in external validation, calibration, reproducibility, implementation and demonstration of clinical utility.

### 3.3. Overview of the Included Evidence Base

The main characteristics of the included studies are summarized in Table 1, with complete study-level extraction reported in Supplementary Table S3. Most studies were retrospective cohort or secondary database analyses. Overall, 65 of 75 studies were retrospective cohort or database-based studies, whereas prospective or prospectively validated evidence remained uncommon. Three studies were classified as prospective or multicenter prospective cohorts, four as retrospective studies with prospective validation, and three as clinical cohort or trial-derived analyses.

Public ICU databases dominated the evidence base. MIMIC-derived data appeared in 69 of 75 studies, including studies using MIMIC-III, MIMIC-IV, unspecified MIMIC versions, or combinations of MIMIC with other datasets. eICU was used in 26 studies, often as an external validation cohort or as part of multi-database model development and testing. Seventeen studies incorporated multicenter or local clinical cohorts, four used omics or transcriptomic data, and one incorporated imaging or multimodal data. This repeated use of MIMIC and eICU facilitated reproducible modeling but also created potential overlap across source populations and limited independence of performance estimates.

Target populations included general adult ICU sepsis or septic shock cohorts, as well as clinically defined subgroups, including sepsis-associated acute kidney injury; sepsis-associated liver injury; sepsis-induced coagulopathy; sepsis-associated delirium or encephalopathy; sepsis-related acute respiratory failure or ARDS; sepsis-induced cardiomyopathy; urosepsis; elderly sepsis; cancer-associated sepsis; diabetes-associated sepsis; postoperative sepsis; sepsis with autoimmune disease; and ICU readmission after sepsis. Sepsis definitions varied across studies and included Sepsis-3 criteria, ICD-coded sepsis, database-derived definitions, and author-defined clinical criteria.

Prediction time zero and feature windows were heterogeneous. Some studies anchored prediction at ICU admission or within the first 24–48 h of ICU stay, while others used sepsis diagnosis, early post-diagnosis windows, dynamic time-series data, organ dysfunction trajectories, treatment trajectories, or subgroup-specific index events. This variability was a major source of heterogeneity and limited direct comparability of model performance across studies.

### 3.4. Prediction Targets and Outcome Domains

Prediction targets are summarized in Table 2. Mortality was the dominant endpoint across the included literature, but outcome definitions and prediction horizons varied substantially. After full-text reassessment, studies were grouped according to their primary or most clinically emphasized prediction target: hospital or in-hospital mortality; ICU mortality; 28-day mortality; 30-day mortality; early or short-term mortality; long-term or survival-type mortality; mortality with unspecified or variable time horizon; composite or multi-outcome prediction; and clinically relevant non-mortality prognostic outcomes.

Hospital or in-hospital mortality and mortality with unspecified or variable horizons were the most frequent categories, with 20 studies each. Fixed-horizon mortality outcomes were also common: nine studies evaluated 28-day mortality, and eight evaluated 30-day mortality. ICU mortality was the primary target in five studies, seven studies evaluated composite or multi-outcome prediction, two focused primarily on early or short-term mortality, two on long-term or survival-type mortality, and two on clinically relevant non-mortality prognostic outcomes. Complete study-level outcome mapping is provided in Supplementary Table S3.

The 28-day mortality domain contained the most clearly defined fixed-horizon external-validation evidence. Clinically informative examples included an XGBoost model for sepsis-induced coagulopathy externally validated in a hospital cohort, with AUROC 0.864 (95% CI, 0.794–0.934), calibration curves, decision-curve analysis and SHAP-based interpretation [27]; and an XGBoost model for sepsis-induced coagulopathy validated across MIMIC-IV and eICU, with AUROC 0.913 and 0.923; AUPRC 0.796 and 0.921; and higher discrimination than SOFA, SAPS II, and SIC score [28]. In septic shock, ShockSurv reported high discrimination in internal/test-cohort evaluation, with AUROC 0.916 in cross-validation and 0.903 in the independent test cohort [30]. In respiratory subgroups, Xu et al. reported XGBoost AUC 0.812 in MIMIC-IV training and 0.714 in eICU external validation for sepsis complicated by acute respiratory failure [29], while Zhang et al. reported external AUROC 0.816 for an SVC model in sepsis complicated by ARDS [33]. In sepsis-associated liver injury, Li et al. reported XGBoost AUROC 0.856 (95% CI, 0.807–0.898) in internal validation, with heterogeneous external performance across MIMIC-III and eICU-CRD, including AUROC 0.880 in MIMIC-III and 0.697 in eICU-CRD [31]. In sepsis complicated by autoimmune diseases, Wang et al. reported external AUROC 0.787 in a small single-center external cohort, with calibration assessment and decision-curve analysis [32]. These studies provide more interpretable evidence than internally validated models with isolated high AUROC values, because they expose both the potential and the transportability limits of AI/ML models across clinically distinct sepsis phenotypes.

Outcome heterogeneity was therefore a central finding rather than a minor methodological inconvenience. Even when the same nominal horizon was used, such as 28-day mortality, studies differed in sepsis phenotype, organ dysfunction subgroup, time zero, feature window, validation cohort, and reporting of uncertainty. Outcome-specific findings were therefore synthesized narratively, and quantitative pooling was not used as the primary synthesis.

### 3.5. Model Families and Data Modalities

Model families and data modalities are summarized in Table 3. Boosting algorithms were the dominant final or clinically emphasized model family, appearing in 44 of 75 studies. These included XGBoost, LightGBM, CatBoost, gradient boosting, and gradient-boosting decision tree approaches, as summarized in Table 3 and detailed in Supplementary Table S3. Their frequent use likely reflects their suitability for structured tabular EHR data and compatibility with post hoc explainability methods such as SHAP.

Random forest or ensemble tree-based approaches were the primary or emphasized family in 12 studies [19,35,49,50,56,57,58,59,60,61,62,63]. Deep-learning approaches, including Transformer-based models, LSTM/time-series models, graph neural networks, and teacher–student architectures, were used in six studies [37,38,51,52,64,65]. Classical machine-learning or SVM-based approaches were the primary family in five studies [33,34,40,53,66]. Seven studies used traditional/statistical models integrated with ML workflows or ML-derived feature selection and benchmarking [32,54,55,67,68,69,70]. One study used a primarily imaging/multimodal AI approach [48].

This distribution shows that the field is still centered on structured EHR-based tabular prediction rather than fully multimodal or prospectively deployed AI systems. Deep-learning, omics-informed, and imaging/multimodal models represented a smaller but methodologically important subset, particularly for dynamic, time-series, or biologically enriched prediction tasks. However, these approaches generally required more complex preprocessing pipelines and were less consistently linked to external validation, calibration, or workflow implementation.

### 3.6. Validation Strategies and Model Performance

Validation strategy was one of the clearest markers separating model development from clinical maturity. External validation was reported in 27 of 75 studies, whereas 48 studies were limited to internal validation, cross-validation, temporal validation, unclear validation, or retrospective database evaluation without clearly independent external testing, as summarized in Table 4 and Table S3. Prospective evaluation or prospectively collected validation evidence was identified in only three studies. Several studies used one public database for model development and another for external validation, most often combinations of MIMIC and eICU. Others used temporal validation, local validation cohorts, or multicenter datasets. However, external validation was not always clearly separated from internal testing, and some studies reported multiple AUROC values across development, internal validation, and external validation cohorts without clearly identifying the clinically proposed estimate.

Discrimination was the dominant performance domain. AUROC or C-statistic was extractable in 64 of 75 studies, as summarized in Table 4 and Table S3. Reported discrimination varied substantially across outcome domains, model families, validation strategies, data sources, and clinical populations. High AUROC values were most often reported in internally validated studies, selected subgroups, or models using highly structured database-derived predictors. By contrast, externally validated models generally showed more variable performance, reinforcing that model performance in development settings should not be assumed to reflect transportability.

Reporting beyond discrimination was less consistent. Calibration was reported in 38 studies, but the depth of assessment ranged from formal calibration plots or Brier scores to brief qualitative statements, as summarized in Table 4 and Table S3. Decision-curve analysis or other clinical utility assessment was reported in 37 studies. AUPRC, threshold-specific performance, net benefit, and clinically interpretable decision thresholds were reported less consistently than AUROC, despite their relevance for imbalanced outcomes and bedside decision-making. Overall, the evidence remains strongly AUROC-centered, and discrimination alone was insufficient to establish clinical readiness.

A small subset of studies provided particularly informative performance evidence because they combined a defined prediction horizon, independent validation, and reporting beyond AUROC. In addition to the sepsis-induced coagulopathy models described above [27,28], respiratory subgroup studies illustrated the importance of external validation for transportability. Xu et al. developed an XGBoost model for 28-day mortality in sepsis complicated by acute respiratory failure using MIMIC-IV and externally validated it in eICU-CRD, with AUC 0.812 in the training cohort and 0.714 in the external validation cohort [29]. Zhang et al. externally validated an SVC model in sepsis complicated by ARDS under the new global ARDS definition, reporting external AUROC 0.816 together with calibration and decision-curve analyses [33]. These studies do not establish generalizability across all ICU sepsis, but they represent the most clinically credible performance signal within the current literature because they combine phenotype-specific outcomes, independent validation, and at least partial assessment beyond discrimination.

### 3.7. Explainability and Model Availability

Explainability or interpretability methods were reported in 64 studies, as summarized in Table 4 and Table S3. SHAP was the most frequently used approach, followed by feature-importance ranking, LIME, partial dependence plots, and model-specific interpretability methods. The most commonly highlighted predictors included age, lactate, organ dysfunction variables, vasopressor exposure, renal indices, coagulation parameters, inflammatory markers, vital signs, and severity scores.

However, explainability was usually implemented as post hoc global feature ranking rather than as patient-level, action-oriented clinical interpretation. Few studies linked explanatory outputs to specific risk thresholds, treatment decisions, clinician behavior or prospective workflow use. In addition, model and code availability were limited; only a minority of studies provided usable online calculators, nomograms, web applications, open-source code, or deployable model objects. Thus, explainability was common, but actionable implementation evidence remained limited.

### 3.8. Comparative Performance Versus Conventional Scores

An important comparative finding was that AI/ML models usually, but not uniformly, achieved discrimination at least comparable to or higher than conventional severity scores in studies that reported direct within-cohort comparisons. Nine studies provided 17 directly extractable cohort- or outcome-specific comparisons between an AI/ML model and a conventional severity score or score-derived comparator, including SOFA, APACHE II, SAPS II, and APS-III [23,28,32,37,38,47,49,51,55]. The individual paired comparisons are reported in Supplementary Table S7. Comparisons reported only graphically or without extractable numeric AUROC values in the full text or uploaded Supplementary Materials were not included in the ΔAUROC calculation. Across these directly extractable paired comparisons, AI/ML models usually showed higher discrimination than the best available conventional comparator, with a median ΔAUROC of +0.108, an interquartile range of +0.082 to +0.148, and a range from −0.013 to +0.203. The direction of effect favored AI/ML models in most comparisons, but not all: in one composite clinical-improvement/chronic-critical-illness comparison, APACHE II slightly outperformed the CatBoost model (ΔAUROC −0.013) [47]. Because paired uncertainty estimates, DeLong confidence intervals and prespecified non-inferiority margins were generally unavailable, these findings should not be interpreted as formal evidence of statistical superiority or non-inferiority.

This finding suggests that AI/ML models can capture prognostic information beyond static severity scores in many settings, particularly when they incorporate high-dimensional EHR data, temporal variables, or nonlinear interactions. However, the comparative signal was not suitable for formal pooling because most studies did not report paired uncertainty estimates, DeLong confidence intervals for AUROC differences, or ROC covariance estimates. Therefore, the model-versus-score finding should be interpreted as a descriptive comparative signal rather than a definitive pooled effect.

**Figure 2:**
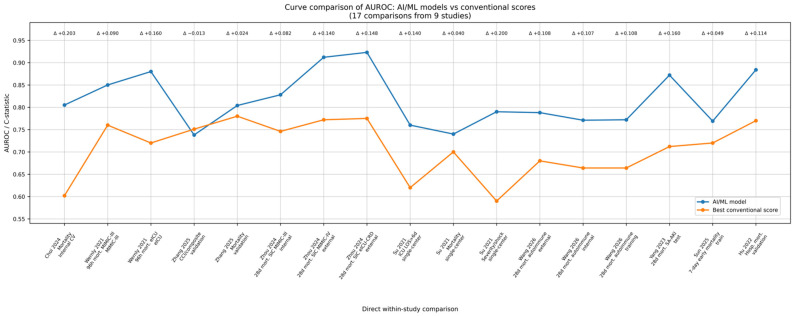
Comparative discrimination of AI/ML models versus conventional severity scores. The figure summarizes 17 direct within-study AUROC/C-statistic comparisons from nine studies [23,28,32,37,38,47,49,51,55]. AI/ML models usually achieved higher discrimination than the best-performing conventional comparator, but the observed ΔAUROC varied substantially, highlighting both the potential incremental value of AI/ML-based prediction and the heterogeneity of model performance across populations, outcomes, and validation settings.

The direct within-study comparison of AUROC/C-statistic values showed that AI/ML models generally achieved higher discrimination than the best-performing conventional severity scores; however, the magnitude of improvement varied across studies and outcomes. As shown in Figure 2, most comparisons favored AI/ML models, with positive ΔAUROC values, but one comparison showed a slight disadvantage, confirming that the superiority of AI/ML approaches was not uniform across all settings.

### 3.9. Risk of Bias and Applicability

Risk-of-bias assessment according to PROBAST domains supplemented by PROBAST + AI considerations is reported in Supplementary Table S4, which provides both study-level judgments and domain-level summary findings. In the main text, we report the overall distribution of risk-of-bias judgments and the main methodological concerns emerging across the participants, predictors, outcome, and analysis domains.

Overall, 45 of 75 studies were judged to be at high risk of bias, while 30 were judged as having some concerns. No study was classified as unequivocally low risk across all domains. The analysis domain was the main driver of high risk of bias. Forty-eight studies were judged to have high concern in this domain, primarily because of internal validation only, possible overfitting, limited or absent sample-size justification, incomplete calibration assessment, unclear feature-selection or hyperparameter-tuning procedures, insufficient reporting of missing-data handling, or lack of robust independent validation.

At the domain level, the participant domain raised concerns when studies relied on highly selected database-derived cohorts, narrow sepsis subgroups, single-center samples, or repeated use of overlapping MIMIC/eICU populations. These issues may limit applicability to broader ICU sepsis populations. The predictors domain was mainly affected by heterogeneous predictor timing, unclear feature windows, and potential predictor–outcome overlap, particularly when organ-support variables, late physiological measurements, or treatment-intensity markers were used to predict mortality. The outcome domain was generally less problematic than the analysis domain, but concerns remained for studies with heterogeneous mortality horizons, unclear time zero, administrative sepsis definitions, or composite endpoints that were not consistently defined across cohorts.

AI-specific concerns were also frequent and included possible data leakage, limited reproducibility, incomplete preprocessing details, unclear hyperparameter tuning, limited code or model availability, and explainability restricted to post hoc feature-importance ranking. Overall, the risk-of-bias assessment indicates that the main limitation of the current evidence base is not the absence of promising discrimination, but the limited methodological robustness required to support reliable clinical translation.

### 3.10. TRIPOD/TRIPOD + AI Reporting Completeness

Reporting completeness according to TRIPOD/TRIPOD + AI domains is reported in Supplementary Table S5, which provides study-level reporting assessments and domain-level summary findings. In the main text, we report the overall reporting pattern and the most recurrent limitations relevant to interpretation, reproducibility, and clinical translation. Several reporting gaps were recurrent across the included studies. Although AUROC or C-statistic was extractable in 64 of 75 studies, calibration was reported in only 38 studies, and decision-curve analysis or another clinical-utility assessment in 37 studies. Prospective evaluation was reported in only three studies. Missing-data handling, predictor timing, feature engineering, hyperparameter tuning, model availability, and code availability were inconsistently described. In particular, many studies provided sufficient information to interpret discrimination but insufficient information to reproduce the full modeling pipeline or assess whether predictions could be implemented as clinically actionable bedside tools.

Overall reporting was classified as good in 41 of 75 studies, while the remaining studies showed moderate or limited completeness, as detailed in Supplementary Table S5. Most studies adequately reported the broad clinical objective, data source, population, and model family. AUROC or C-statistic was commonly reported, with extractable values in 64 studies.

However, important reporting limitations were recurrent. Outcome definitions and prediction horizons were not always explicit. Prediction time zero and feature windows were sometimes unclear. Missing-data mechanisms and imputation strategies were incompletely described in several reports. Hyperparameter tuning, preprocessing pipelines, and model-selection procedures were variably reported. Calibration, decision-curve analysis, clinical utility, and model availability were less consistently reported than discrimination. Explainability was frequently included but often presented as global feature ranking rather than as clinically integrated interpretation.

Few studies reported sufficient information to enable independent model reproduction, prospective validation, or direct implementation in ICU workflows. Overall, TRIPOD/TRIPOD + AI findings indicated that reporting quality was stronger for discrimination and general model description than for calibration, clinical utility, reproducibility, and implementation readiness.

### 3.11. Translational Readiness of AI/ML Models

To further clarify the clinical maturity of the included evidence, translational readiness criteria were summarized across studies (Table 4). These criteria were selected because they represent key steps between retrospective model development and clinically usable bedside decision support: external validation, prospective evaluation, calibration, decision-curve analysis or clinical-utility assessment, explainability, comparison with conventional severity scores, model or code availability, and workflow-level implementation.

Overall, this translational-readiness summary indicates that the current literature is more mature in retrospective discrimination reporting than in clinical implementation. Although many models reported promising AUROC values and most included some form of explainability, fewer studies reported external validation, calibration, decision-curve analysis, or prospective evaluation. Therefore, the available evidence supports the methodological promise of AI/ML-based prediction in adult ICU sepsis, but it does not yet establish broad readiness for routine bedside deployment.

### 3.12. Quantitative Synthesis Feasibility

Formal meta-analysis was not performed as the primary synthesis. Although several studies reported AUROC or C-statistic values, no outcome cluster provided a sufficiently homogeneous set of independent studies with comparable population, prediction time horizon, validation setting, model purpose, and extractable uncertainty estimates. In addition, repeated use of overlapping public ICU databases, particularly MIMIC and eICU, limited the assumption of independent performance estimates across studies.

The 28-day mortality domain was the closest candidate for exploratory quantitative synthesis because several externally validated AUROC estimates were available [27,28,29,30,31,32,33]. However, these studies addressed clinically distinct subgroups, including sepsis-induced coagulopathy, septic shock, sepsis-associated liver injury, autoimmune disease-associated sepsis, acute respiratory failure, and ARDS, and they differed in model architecture, validation source, and reporting completeness. They also showed variable transportability across databases; for example, external validation in sepsis-associated liver injury ranged from AUROC 0.880 in MIMIC-III to 0.697 in eICU-CRD [31]. Because of this clinical and methodological heterogeneity, quantitative pooling was not retained as a primary result, and the evidence was synthesized narratively.

Similarly, a meta-analysis of AI/ML versus conventional score differences was not performed. Although 17 directly extractable paired comparisons from nine studies were identified, most did not report paired ΔAUROC uncertainty, DeLong confidence intervals for the difference, or ROC covariance. Comparative performance was therefore reported descriptively.

## 4. Discussion

### 4.1. Principal Findings

This systematic review shows that AI-, ML-, and DL-based outcome prediction in adult ICU sepsis has moved beyond proof-of-concept modeling, but has not yet reached clinical implementation readiness. Across 75 included studies, discrimination was commonly reported, with AUROC or C-statistic extractable in 64 studies, as summarized in Table 4 and Table S3. A clinically relevant comparative signal also emerged: across 17 directly extractable cohort- or outcome-specific comparisons from nine studies, AI/ML models usually, but not uniformly, achieved higher discrimination than conventional severity scores, with a median paired ΔAUROC of +0.108 (IQR, +0.082 to +0.148; range, −0.013 to +0.203) [23,28,32,37,38,47,49,51,55]. These findings support the premise that data-driven models can capture prognostic information not fully represented by static acuity scores, while also showing that conventional scores remain competitive for selected endpoints and cohorts.

At the same time, the evidence base demonstrates a clear translational gap. External validation was reported in only 27 studies, prospective evaluation in 3, calibration in 38, and decision-curve analysis or another clinical utility assessment in 37, as summarized in Table 4 and Table S3. Moreover, 45 studies were judged to be at high risk of bias, mainly because of limitations in the analysis domain. The central message is therefore balanced: the field shows a meaningful predictive signal, but most published models remain insufficiently validated, calibrated, reproducible, and operationalized for bedside decision support. A central finding of this review is that heterogeneity was not limited to model architecture, but involved almost every clinically relevant dimension of prediction modeling. Included studies differed in sepsis definitions, population selection, ICU setting, data source, prediction time zero, feature window, outcome horizon, validation strategy, and performance reporting. This heterogeneity was particularly evident across outcome domains: ICU mortality, in-hospital mortality, 28-day mortality, 30-day mortality, long-term mortality, organ dysfunction, readmission, and composite outcomes addressed different clinical questions and were not directly interchangeable. Therefore, the results should be interpreted as an evidence map of AI/ML prognostic modeling in adult ICU sepsis rather than as a single homogeneous estimate of model performance.

### 4.2. Contribution of This Review

A key contribution of this review is that it separates apparent algorithmic performance from clinically credible evidence. Rather than treating all AUROC values as equivalent, we distinguished internally validated models from externally validated models, fixed-horizon endpoints from heterogeneous outcome definitions, and direct model-versus-score comparisons from non-comparable performance estimates. This approach shows that the AI/ML literature in ICU sepsis should not be viewed as merely exploratory: several models demonstrate clinically meaningful discrimination, and some outperform established severity scores in direct comparisons. At the same time, the review clarifies why this promising signal has not yet translated into bedside implementation. The key question is no longer whether AI/ML can predict outcomes in ICU sepsis, but whether these predictions are sufficiently calibrated, transportable, interpretable, and actionable to improve clinical decisions.

### 4.3. Clinical Meaning of AI-Based Prediction in ICU Sepsis

Sepsis is a dynamic syndrome in which prognosis depends on baseline vulnerability, infection source, immune-inflammatory response, organ dysfunction trajectories, treatment timing, and ICU exposures [1,2]. This complexity provides a strong rationale for AI/ML-based prognostic modeling. Unlike static severity scores, AI/ML models can incorporate high-dimensional EHR variables; nonlinear interactions; and, in selected studies, time-series or multimodal data structures, as detailed in Supplementary Table S3. These features may explain the recurrent discrimination advantage observed in studies comparing AI/ML models with conventional scores.

Nevertheless, clinical risk prediction in ICU sepsis requires more than ranking patients by risk. A model intended for bedside decision support should provide calibrated absolute probabilities, remain stable across hospitals, identify clinically meaningful thresholds, and support decisions that can plausibly improve care. This distinction is essential. An AUROC advantage may be statistically attractive but clinically insufficient if the model is poorly calibrated, externally unvalidated, unavailable for implementation, or disconnected from a specific decision pathway.

### 4.4. AI/ML Models Versus Conventional Severity Scores

The comparison between AI/ML models and conventional severity scores is one of the most clinically informative findings of this review. Conventional scores such as SOFA, qSOFA, APACHE, SAPS, LODS, and OASIS remain useful for organ dysfunction assessment, severity description, benchmarking, and population-level risk stratification [3,4,5,6,7,8]. However, they were not designed to capture all nonlinear, time-varying, and high-dimensional patterns relevant to individualized prognosis in heterogeneous ICU sepsis populations.

In studies with directly extractable within-cohort comparisons, AI/ML models usually achieved higher or at least comparable discrimination relative to the best available conventional comparator [23,28,32,37,38,47,49,51,55]. However, this finding should be interpreted cautiously and should be considered a descriptive signal rather than definitive evidence of clinical superiority. The advantage was not universal: one comparison slightly favored APACHE II over CatBoost for a composite clinical-improvement/chronic-critical-illness endpoint [47]. In addition, the available comparisons primarily addressed discrimination, not calibration, decision-analytic benefit, clinical usefulness, or workflow-level implementation. Because most studies did not report paired uncertainty estimates, DeLong confidence intervals for AUROC differences or ROC covariance estimates, and because no non-inferiority or superiority margin was prespecified, the model-versus-score comparison cannot be interpreted as proof of statistical superiority, formal non-inferiority, or readiness to replace conventional severity scores in routine practice. Rather, AI/ML models should be viewed as potentially complementary prognostic tools that require stronger external validation, calibration assessment, prospective evaluation, and clinical-utility testing before bedside adoption.

### 4.5. Most Clinically Informative Studies

The strongest current evidence does not come from the highest AUROC values alone, but from studies combining clinically defined outcomes; independent validation; and at least partial assessment of calibration, clinical utility, or interpretability. In sepsis-induced coagulopathy, Wu et al. reported external hospital validation of an XGBoost model for 28-day mortality, with AUROC 0.864 (95% CI, 0.794–0.934), calibration curves, DCA, and SHAP interpretation [27]. Zhou et al. reported XGBoost performance across MIMIC-IV and eICU, with AUROC 0.913 and 0.923, and AUPRC 0.796 and 0.921, respectively, and higher discrimination than SOFA, SAPS II, and SIC score [28].

Other clinically informative examples showed more variable transportability across sepsis phenotypes. In septic shock, ShockSurv reported high test-cohort discrimination, with AUROC 0.903 in the independent test cohort [30]. In sepsis complicated by acute respiratory failure, Xu et al. reported external validation in eICU with AUC 0.714 [29], while Zhang et al. reported external AUROC 0.816 for an SVC model in sepsis complicated by ARDS [33]. In sepsis-associated liver injury, Li et al. reported AUROC 0.856 in internal validation, but external validation varied substantially across MIMIC-III and eICU-CRD [31]. In autoimmune disease-associated sepsis, Wang et al. reported external AUROC 0.787 in a small independent cohort, with calibration and DCA [32]. These studies are more informative than isolated high-AUROC internally validated models because they better expose the central issue for clinical translation: performance can be promising within specific phenotypes, but transportability remains variable.

### 4.6. Validation and Calibration Remain the Main Translational Bottlenecks

The most important limitation of the field is the imbalance between model development and model validation. Most studies were retrospective and database-based, with heavy reliance on MIMIC-derived data and eICU. These databases have enabled reproducible research, transparent benchmarking, and large-scale model development, but repeated reuse of overlapping or closely related ICU populations may reduce the true independence of the evidence base. Models trained and tested within public ICU repositories may reflect database-specific coding practices, measurement frequency, missing-data mechanisms, treatment protocols, case-mix distribution, and healthcare-system characteristics. Consequently, performance in MIMIC or eICU should not be assumed to translate automatically to other hospitals, countries, electronic health record systems, or ICU workflows. Independent external validation in geographically, institutionally, and temporally distinct cohorts remains essential before clinical deployment.

External validation was reported in fewer than half of the included studies, and prospective evaluation was rare. This substantially limits confidence in clinical transferability. In ICU sepsis, where case-mix, microbiology, organ support strategies, and practice patterns differ across institutions, external validation is not optional. It is a prerequisite for clinical credibility.

Another major translational gap concerns the imbalance between discrimination reporting and clinically meaningful evaluation. AUROC was the dominant metric, but AUROC alone is insufficient for bedside decision support. Calibration was reported in only 38 studies, and the depth of assessment varied widely. This is a critical gap because clinical decisions require reliable absolute risk estimates, not only discrimination. A model may correctly rank patients but still overestimate or underestimate mortality risk. Such miscalibration could affect escalation decisions, triage, family communication, resource allocation, or trial enrichment. For a prognostic model to guide individualized care, predicted risks must be well calibrated, clinically actionable thresholds must be defined, and net benefit should be evaluated across plausible decision thresholds. Decision-curve analysis, calibration plots, Brier scores, and threshold-specific performance were inconsistently reported, and prospective workflow-level evaluation was rare. This limits the ability to determine whether a model would improve clinical decisions, reduce unnecessary interventions, support timely escalation or de-escalation, or improve patient-centered outcomes. The translational-readiness summary reported in Table 4 further shows that the current evidence base is more mature in retrospective discrimination reporting than in external validation, calibration, prospective testing, model availability, and workflow-level implementation.

### 4.7. Clinical Utility and Implementation Evidence Remain Limited

Decision-curve analysis or another clinical utility assessment was reported in 37 studies, as summarized in Table 4 and Table S3. This represents progress compared with purely discrimination-based reporting, but most studies still did not clearly link model thresholds to specific bedside decisions. For an ICU sepsis prediction model to be clinically useful, it should answer an actionable question: whether to intensify monitoring, trigger senior review, modify organ support, enroll a patient in a trial, reassess goals of care, or allocate resources differently.

Without this decision link, even technically strong models remain difficult to interpret clinically. Future studies should move beyond reporting AUROC and SHAP plots and toward threshold-based evaluation, net benefit, workflow consequences, and prospective impact assessment. A clinically useful model is not simply one that predicts well; it is one that improves or rationalizes decisions when used in real care pathways.

### 4.8. Explainability Is Common but Often Not Actionable

Explainability was reported in 64 studies, most commonly through SHAP, feature-importance ranking, LIME, or related post hoc methods, as summarized in Table 4 and Table S3. This is encouraging because transparency is particularly important in critical care, where black-box predictions may be difficult to trust or operationalize. The predictors highlighted by explainability analyses were often clinically plausible, including age, lactate, organ dysfunction variables, vasopressor exposure, renal indices, coagulation markers, inflammatory markers, vital signs, and severity scores.

However, explainability should not be equated with actionability. Most explanations were global descriptions of model behavior rather than patient-level decision tools. Few studies showed how explanations would modify clinician behavior, reduce uncertainty, or improve outcomes. Moreover, feature importance does not establish causality. In sepsis, variables such as vasopressor dose, lactate, mechanical ventilation, or renal dysfunction may reflect severity, treatment response, confounding by indication, or disease trajectory. Explainability is therefore necessary for transparency but insufficient as evidence of clinical usefulness.

### 4.9. Risk of Bias and Reporting Quality

The PROBAST/PROBAST + AI assessment showed frequent methodological concerns, with 45 of 75 studies judged at high risk of bias, as detailed in Supplementary Table S4 [15,16]. The analysis domain was the main driver. Common problems included internal validation only, possible overfitting, limited sample-size justification, incomplete calibration, unclear hyperparameter tuning, incomplete missing-data reporting, and potential data leakage. These limitations directly affect the credibility of reported model performance.

TRIPOD/TRIPOD + AI reporting assessment also identified recurrent gaps [17,18]. Most studies described their objective, data source, population, and model family, and AUROC was commonly reported. However, reporting was weaker for prediction time zero, feature windows, missing-data handling, preprocessing, tuning, calibration, clinical utility, model availability, and implementation readiness. This pattern is important because incomplete reporting limits replication, independent validation, and clinical translation. Future studies should adopt TRIPOD + AI prospectively during study design and manuscript preparation rather than using it retrospectively as a reporting checklist.

### 4.10. Quantitative Synthesis and Interpretability of Performance Estimates

Formal quantitative synthesis was not retained as the primary summary of evidence because the included studies were too heterogeneous in population, outcome horizon, prediction time zero, model type, validation setting, and uncertainty reporting. This was not merely a statistical inconvenience; it reflected genuine clinical and methodological diversity. ICU mortality, in-hospital mortality, 28-day mortality, 30-day mortality, and long-term mortality are not interchangeable endpoints. Similarly, general ICU sepsis, septic shock, sepsis-induced coagulopathy, sepsis-associated liver injury, sepsis-associated acute kidney injury, and autoimmune disease-associated sepsis represent different clinical contexts.

The 28-day mortality domain was the closest candidate for quantitative synthesis because several externally validated AUROC estimates were available [27,28,29,30,31,32,33]. However, these studies represented different clinical subgroups, including sepsis-induced coagulopathy, septic shock, sepsis-associated liver injury, autoimmune disease-associated sepsis, acute respiratory failure, and ARDS, and they differed in model architecture, validation source, and reporting completeness. Transportability was also variable across external validation sources, as illustrated by the liver-injury model that performed well in MIMIC-III but substantially less well in eICU-CRD [31]. Pooling such studies as if they reflected a single homogeneous population would risk producing a misleading estimate of model performance. For this reason, quantitative pooling was not retained as the primary summary of evidence.

The same principle applied to AI/ML-versus-score comparisons. Although the descriptive direction of effect favored AI/ML models in most direct comparisons, paired uncertainty was not consistently reported, and one comparison favored a conventional score. A formal meta-analysis of ΔAUROC would therefore require assumptions not supported by the published data. Descriptive synthesis was the more rigorous and transparent choice.

### 4.11. Sepsis Heterogeneity and Future Multimodal Prediction

Sepsis is not a single disease but a heterogeneous syndrome characterized by variable host responses, pathogen profiles, infection sources, organ dysfunction patterns, immune-inflammatory states, treatment exposures, and recovery trajectories. This heterogeneity was reflected in the included studies, which evaluated general adult ICU sepsis cohorts, as well as subgroups such as septic shock, sepsis-induced coagulopathy, sepsis-associated acute kidney injury, liver injury, delirium or encephalopathy, acute respiratory failure, autoimmune disease-associated sepsis, and cancer-associated sepsis, as detailed in Supplementary Table S3. A model trained in one sepsis phenotype may not be valid in another. Similarly, a mortality model for septic shock may not be directly comparable with a model for sepsis-associated liver injury, sepsis-induced coagulopathy, or post-ICU readmission.

The need for better phenotyping is also supported by emerging critical-care studies outside the AI prediction model corpus. Bruni et al. showed that lung microbiota composition differed in pneumonia patients across ICU and non-ICU settings, suggesting that microbiome-derived phenotyping may provide additional insight into pulmonary infection biology and dysbiosis [71]. Similarly, invasive fungal infections during ECMO represent a high-risk example of how infection, extracorporeal support, antimicrobial exposure, colonization, organ failure, and pharmacokinetic complexity interact in critically ill patients. Serapide et al. reported invasive fungal infections during extracorporeal membrane oxygenation as a clinically complex ICU scenario with very high mortality among affected patients [72]. These contextual studies were not included in the AI/ML prognostic model synthesis, but they illustrate why future sepsis prediction models may need to move beyond generic mortality classification and toward biologically informed, temporally updated, and clinically actionable phenotypes.

### 4.12. Implications for Future Research

The next phase of AI research in ICU sepsis should shift from algorithm development to clinical validation. Future studies should define prediction time zero, feature windows, and outcome horizons before model development. External validation should be performed in independent, clinically distinct cohorts whenever possible. Calibration should be reported systematically, including calibration plots and quantitative metrics. Decision-curve analysis should be linked to explicit bedside thresholds and clinical actions.

AI-specific methodological risks should be addressed prospectively. These include temporal leakage, predictor-outcome overlap, overfitting during model selection, inadequate handling of missingness, dataset shift, and lack of reproducibility. Studies using public ICU databases should provide cohort definitions, extraction logic, preprocessing code, and model specifications in enough detail to enable replication. When feasible, investigators should share code, trained model objects or deployable calculators.

Most importantly, future work should test whether AI/ML prediction improves care. Prospective workflow-integrated studies are needed to determine whether sepsis prognostic models can improve triage, monitoring, escalation, trial enrichment, resource allocation, or communication without increasing alert fatigue, bias, or inappropriate treatment decisions.

### 4.13. Strengths and Limitations of This Review

This review has several strengths. It focused specifically on adult ICU sepsis; incorporated PubMed/MEDLINE, Scopus, and Cochrane Library searches up to April 2026; applied adult-only refinement; and included 75 studies. It evaluated not only discrimination but also validation, calibration, clinical utility, explainability, comparative performance versus conventional scores, risk of bias, and reporting completeness. A further strength is the conservative handling of comparative performance: the ΔAUROC analysis was restricted to directly extractable within-study comparisons using the same cohort, outcome and validation setting, thereby avoiding misleading cross-study comparisons between non-equivalent models and populations. The use of PRISMA, PROBAST/PROBAST + AI, and TRIPOD/TRIPOD + AI frameworks strengthened the methodological appraisal [14,15,16,17,18].

Several limitations should also be acknowledged. First, the review protocol was not prospectively registered. This may have increased the risk of selective methodological decisions and should be acknowledged as a limitation under PRISMA 2020. However, this risk was mitigated by transparent reporting of the complete search strategies, eligibility criteria, study-selection flow, full-text exclusion categories, extraction fields, risk-of-bias assessment, reporting-completeness domains, and synthesis rules in the Methods section and Supplementary Materials. Second, the included studies were highly heterogeneous in population, data source, sepsis definition, prediction time zero, feature window, model architecture, outcome domain, and prediction horizon. This heterogeneity limited direct comparability across studies and precluded formal meta-analysis. Third, many studies relied on overlapping public ICU databases, particularly MIMIC and eICU, limiting the independence and generalizability of the evidence base. Fourth, reporting was incomplete for several key items, including missing-data handling, calibration, tuning procedures, model availability, and code availability. Fifth, meta-analysis was not used as the primary synthesis because of heterogeneity and insufficient uncertainty reporting. Sixth, the descriptive ΔAUROC analysis was deliberately restricted to within-study comparisons with numeric AUROC/C-statistic values directly extractable from the full-text articles and available Supplementary Materials; comparisons reported only graphically, incompletely, or in unavailable supplementary/source-data files were not included. Finally, although full texts were reviewed, some performance details could not be extracted consistently because of variable reporting across studies.

## 5. Conclusions

This systematic review shows that AI-, ML-, and DL-based prognostic models in adult ICU sepsis have evolved from exploratory algorithm development into a substantial and clinically relevant evidence base. Across 75 included studies, most models reported measurable discrimination, and directly extractable within-study comparisons showed that AI/ML models often achieved discrimination comparable to or higher than conventional severity scores. However, this finding should be interpreted as a descriptive signal rather than definitive evidence of clinical superiority, because the available evidence remains clinically and methodologically heterogeneous, predominantly retrospective, strongly dependent on MIMIC/eICU-derived data, incompletely calibrated, insufficiently prospectively validated, and frequently at high risk of bias.

The most compelling evidence was observed in externally validated, phenotype-specific models, particularly in sepsis-induced coagulopathy, septic shock, acute respiratory failure, ARDS, liver injury, and autoimmune disease-associated sepsis. These studies suggest that AI-assisted prognostication may be most useful when applied to clearly defined clinical contexts rather than to sepsis as a single homogeneous syndrome.

However, the field has not yet reached routine clinical implementation readiness. The main barrier is not the absence of predictive signal, but the limited demonstration of calibration, transportability, reproducibility, clinical utility, and prospective workflow-level impact. Future research should move beyond retrospective AUROC optimization and prioritize externally validated, calibrated, reproducible, interpretable, and clinically actionable models embedded in prospective ICU workflows. The next step for sepsis AI is not simply to build more models, but to determine which models improve clinical decision-making, for which patients, at which time point, against which clinical benchmark, and under which implementation conditions.

## Figures and Tables

**Table 1:** General characteristics of included studies.

Domain	Summary of Findings	Interpretation for Synthesis
Included studies	75 studies were included in the final qualitative synthesis.	Broad evidence base, but clinically and methodologically heterogeneous.
Source of included studies	50 studies were PubMed-derived and 25 were additional Scopus-derived studies.	The Scopus search substantially expanded the adult ICU corpus.
Study design	65/75 were retrospective cohort or database-based studies; prospective or prospectively validated evidence was uncommon.	Evidence mainly reflects secondary-data modeling rather than prospective bedside implementation.
Data sources	MIMIC-derived data appeared in 69/75 studies; eICU in 26/75; local or multicenter cohorts in 17/75; omics/transcriptomics in 4/75; imaging/multimodal data in 1/75.	Reuse of public ICU databases improves accessibility but raises concerns about overlap, transportability, and independence of estimates.
Populations	Studies included general ICU sepsis/septic shock cohorts and subgroups such as SA-AKI, SIC, SALI, ARDS/acute respiratory failure, SAE/delirium, cancer, diabetes, urosepsis, autoimmune disease, and postoperative sepsis.	Clinical heterogeneity limits direct comparison and supports structured narrative synthesis.
Prediction timing	Time zero varied across ICU admission, sepsis diagnosis, early ICU windows, dynamic trajectories, and subgroup-specific index events.	Inconsistent prediction timing was a key limitation for interpretation and applicability.
Performance reporting	AUROC/C-statistic was extractable in 64/75 studies.	Discrimination was the dominant metric, but not sufficient to establish clinical readiness.
Validation	External validation was reported in 27/75 studies; prospective evaluation in 3/75.	External and prospective validation remained limited relative to model-development activity.

The complete list of included studies is provided in Supplementary Table S3. References to individual studies are reported in the main text only when specific representative examples are discussed. Abbreviations: AUROC, area under the receiver operating characteristic curve; eICU, eICU Collaborative Research Database; ICU, intensive care unit; MIMIC, Medical Information Mart for Intensive Care; SA-AKI, sepsis-associated acute kidney injury; SAE, sepsis-associated encephalopathy; SALI, sepsis-associated liver injury; SIC, sepsis-induced coagulopathy. Footnote: Study characteristics were summarized after full-text review of the 75 included studies. Data-source categories are not mutually exclusive because some studies used more than one database or combined public ICU databases with local, multicenter, omics, imaging or multimodal cohorts. “MIMIC-derived data” includes studies using MIMIC-III, MIMIC-IV, unspecified MIMIC versions, or combinations of MIMIC with other datasets. “External validation” refers to validation in an independent database, institution, temporal cohort, or geographically distinct cohort, as reported by the original authors.

**Table 2:** Outcome-specific evidence profile of AI/ML prediction models in adult ICU sepsis.

Outcome Domain	*n*	Key Examples	AUROC/C-Statistic	Ext. Val.	Calib.	DCA/Utility	Explain.	High ROB
Hospital/in-hospital mortality	20	Selected examples with in-hospital/hospital mortality modeling include [19,20,21,22,23].	16/20	6/20	12/20	7/20	15/20	12/20
ICU mortality	5	Selected ICU mortality examples include cancer-, hypoalbuminemia-, and encephalopathy-focused cohorts [24,25,26].	5/5	4/5	3/5	4/5	5/5	1/5
28-day mortality	9	Most informative fixed-horizon examples include SIC, acute respiratory failure, septic shock, SALI, autoimmune sepsis, and ARDS cohorts [27,28,29,30,31,32,33].	7/9	6/9	6/9	7/9	9/9	3/9
30-day mortality	8	Examples include general Sepsis-3 and subgroup-specific 30-day mortality models [34,35,36].	8/8	2/8	5/8	3/8	7/8	6/8
Early/short-term mortality	2	Examples include routinely available ABG-based and early post-diagnosis models [37,38].	2/2	1/2	0/2	1/2	½	1/2
Long-term mortality	2	Examples include short/long-term prediction and survival-oriented modeling [39,40].	2/2	0/2	1/2	1/2	½	2/2
Mortality, unspecified/variable horizon	20	Selected examples include risk-stratification, phenotype-based, and interpretable mortality models [41,42,43,44,45].	16/20	5/20	7/20	11/20	18/20	15/20
Composite/multi-outcome prediction	7	Examples include multi-prognosis, severity, ICU length of stay, and adverse-event prediction [46,47,48,49].	7/7	2/7	2/7	1/7	6/7	4/7
Clinically relevant non-mortality outcomes	2	Heterogeneous non-mortality or diagnosis-plus-outcome endpoints were retained for narrative synthesis; complete mapping is provided in Supplementary Table S3.	1/2	1/2	2/2	2/2	2/2	1/2

Abbreviations: AUROC, area under the receiver operating characteristic curve; Calib., calibration; DCA, decision-curve analysis; Ext. val., external validation; Explain., explainability or interpretability reporting; ICU, intensive care unit; ROB, risk of bias. Footnote: Studies were classified according to the primary or most clinically emphasized prediction target after full-text review. Secondary outcomes were retained in Supplementary Table S3 but were not double-counted in this table. Key examples are illustrative and not exhaustive; the complete study-level outcome mapping is provided in Supplementary Table S3. “High ROB” indicates studies judged at high overall risk of bias according to PROBAST domains supplemented by PROBAST + AI considerations.

**Table 3:** AI/ML model families, data modalities, and translational implications.

Model Family/Data Type	*n*	Selected Examples	Representative Approaches	Main Use	Methodological Implication
Boosting models	44	[23,27,28,29,30,31,33]	XGBoost, LightGBM, CatBoost, GBM, GBDT	Mortality prediction in general and subgroup cohorts	Most frequent final/emphasized family; strong discrimination was often reported, but external validation and calibration remained variable.
RF/ensemble ML	12	[19,35,49,50]	Random forest, ensemble trees, stacked models	Mortality, severity, adverse prognosis	Useful benchmark/final model family; transportability and calibration remained inconsistent.
Deep-learning/temporal models	6	[37,38,51,52]	ANN, LSTM, Transformer, GNN, teacher–student models	Dynamic mortality, early deterioration, complex EHR modeling	Higher complexity requires stronger reproducibility, external validation, and interpretability.
SVM/classical ML	5	[33,40,53]	SVM, kNN, Naive Bayes, decision trees, regularized ML; transcriptomic SVM	Mortality and subgroup prognosis	Often used as comparator or in smaller datasets; some specialized data modalities were modeled with classical ML.
Statistical + ML hybrids	7	[32,54,55]	Regression + ML feature selection, nomograms, ML-benchmarked models	Mortality and subgroup prognosis	May improve interpretability, but still requires external validation and calibration.
Imaging/multimodal AI	1	[48]	Skin spectral imaging/multimodal AI	Diagnosis plus outcome prediction	Promising but limited by special data requirements and uncertain generalizability.

Abbreviations: ANN, artificial neural network; EHR, electronic health record; GBM, gradient-boosting machine; GBDT, gradient-boosting decision tree; GNN, graph neural network; kNN, k-nearest neighbors; ML, machine learning; RF, random forest; SVM, support vector machine. Footnote: Studies were classified according to the final, best-performing, or clinically emphasized model family after full-text review. Many studies tested multiple algorithms; therefore, model-family categories reflect the primary model used for synthesis rather than all algorithms evaluated within each study. Selected examples are illustrative; complete study-level model mapping is provided in Supplementary Table S3. Specialized data modalities, such as transcriptomic or imaging data, were classified according to the primary modeling approach when the final model was not itself a multimodal architecture.

**Table 4:** Translational readiness criteria across included AI/ML prognostic models.

Translational Criterion	Findings Across Included Studies	Interpretation
External validation	27/75 studies	Present in a minority of studies; essential for assessing transportability across institutions, countries, coding systems, and ICU workflows.
Prospective evaluation	3/75 studies	Rare; the evidence base remains dominated by retrospective model development and validation.
Calibration assessment	38/75 studies	Inconsistently reported; limits interpretation of absolute risk estimates and clinical risk stratification.
Decision-curve analysis or clinical-utility assessment	37/75 studies	Frequently absent or not linked to clinically actionable thresholds.
Explainability or interpretability	64/75 studies	Common, but usually post hoc and rarely connected to patient-level decisions or prospective workflow use.
Direct comparison with conventional severity scores	17 comparisons from 9 studies	Suggests a recurrent but non-uniform discrimination advantage of AI/ML models; not sufficient to prove clinical superiority.
Model or code availability	Inconsistently reported	Limits reproducibility, independent testing, external validation, and implementation.
Workflow-level implementation	Rare or not clearly reported	Current evidence supports methodological promise more than routine bedside deployment.

Criteria were extracted from full-text review and Supplementary Tables S3 and S5. Categories are not mutually exclusive. External validation includes independent database, institutional, temporal, or geographically distinct validation as reported by the original authors. Direct comparisons with conventional severity scores refer only to comparisons with extractable AUROC/C-statistic values for both the AI/ML model and the conventional comparator within the same study, outcome, and validation setting. AI, artificial intelligence; ICU, intensive care unit; ML, machine learning.

## Data Availability

The data supporting the findings of this study are available within the article and its Supplementary Materials. Further inquiries can be directed to the corresponding author.

## Supplementary Materials

The following supporting information can be downloaded at https://www.mdpi.com/article/10.3390/jpm16070346/s1, Supplementary Table S1: Full database search strategies; Supplementary Table S2: Full-text screening and exclusion log; Supplementary Table S3. Study-level extraction summary of included studies; Supplementary Table S4: Study-level PROBAST/PROBAST+AI assessment; Supplementary Table S5: TRIPOD/TRIPOD+AI reporting assessment; Supplementary Table S6: Quantitative synthesis feasibility matrix; Supplementary Table S7: Directly extractable AI/ML versus conventional severity-score comparisons; Supplementary Table S8: PRISMA 2020 checklist.
