# Supplementary material for: Artificial Intelligence Models for Mortality and Outcome Prediction in Intensive Care Unit Sepsis: A Systematic Review
Source: J Pers Med. 2026 Jun 25;16(7):346. doi: 10.3390/jpm16070346 (PMC13412256; doi:10.3390/jpm16070346)
Supplement: Supplementary file 1 [file jpm-16-00346-s001.zip › jpm-4376718-supplementary.pdf]

Supplementary Materials

Artificial Intelligence Models for Mortality and Outcome Prediction in Intensive Care Unit Sepsis: A Systematic Review

These supplementary materials accompany the revised manuscript. The complete list of the 75 included AI/ML studies is provided in Supplementary Table S3. References in the revised main manuscript have been renumbered after removal of included-study references not cited individually in the main text; therefore, the numbering used in Supplementary Table S3 is intended only for the supplementary extraction table and does not represent active citation numbering in the revised main manuscript.

Supplementary Table S1. Full database search strategies

Searches were performed up to April 2026. Database-specific strings and filters are summarized below.

Database	Search date	Search strategy	Filters / limits	Records retrieved / contribution
PubMed/MEDLINE	April 2026	(("sepsis"[MeSH Terms] OR sepsis[tiab] OR "septic shock"[tiab]) AND ("Intensive Care Units"[MeSH Terms] OR "intensive care"[tiab] OR ICU[tiab] OR "critical care"[tiab]) AND ("Artificial Intelligence"[MeSH Terms] OR "Machine Learning"[MeSH Terms] OR "artificial intelligence"[tiab] OR "machine learning"[tiab] OR "deep learning"[tiab] OR "neural network*"[tiab] OR XGBoost[tiab] OR "random forest"[tiab] OR "predictive model*"[tiab]) AND (mortality[tiab] OR prognosis[tiab] OR outcome*[tiab] OR prediction[tiab] OR risk[tiab]))	Humans; English language; article type; publication within the last 10 years	1055 records initially identified; 726 records remained after filters; 50 adult ICU studies included
Scopus	April 2026	TITLE-ABS-KEY((sepsis OR "septic shock") AND ("intensive care" OR ICU OR "critical care") AND ("artificial intelligence" OR "machine learning" OR "deep learning" OR "neural network" OR XGBoost OR "random forest" OR "predictive model") AND (mortality OR prognosis OR outcome OR prediction OR risk))	Duplicates/already PubMed-included records, pediatric/neonatal studies, non-ICU populations and non-AI/ML prognostic studies excluded during screening	223 records identified; 37 full-text reports assessed; 25 additional adult ICU studies included
Cochrane Library	April 2026	sepsis OR septic shock AND artificial intelligence OR machine learning OR deep learning AND prediction OR prognosis OR mortality	No additional restrictions beyond database-specific search options	No additional eligible adult ICU sepsis AI/ML prognostic prediction studies identified

Abbreviations: AI, artificial intelligence; ICU, intensive care unit; MeSH, Medical Subject Headings; ML, machine learning; TITLE-ABS-KEY, Scopus title/abstract/keyword search field.

Footnote: Search dates, search strings and filters are reported as implemented. PubMed counts reflect records before and after database filters; Scopus counts reflect additional screening after duplicate or already-included records were handled.

Supplementary Table S2. Full-text screening and exclusion log

Source	Stage	No. excluded	Main reasons for exclusion
PubMed/MEDLINE	Title/abstract screening	594	Not adult ICU sepsis; not AI/ML; not prognostic outcome prediction; diagnostic-only studies;

Source	Stage	No. excluded	Main reasons for exclusion
			reviews/editorials/commentaries; irrelevant populations or outcomes
PubMed/MEDLINE	Full-text assessment and adult-only refinement	82	Not eligible after full-text assessment, n = 78; pediatric studies, n = 2; full text not retrievable, n = 2
Scopus	Before full-text assessment	186	Duplicate or already PubMed-included records; pediatric/neonatal studies; non-ICU populations; non-sepsis populations; diagnostic-only studies; not AI/ML-based prognostic prediction
Scopus	Full-text assessment	12	Duplicate corpus; pediatric/neonatal population; non-ICU population; not primarily adult ICU sepsis; not AI/ML-based prognostic outcome prediction
Cochrane Library	Eligibility check	0 additional included	No additional eligible adult ICU sepsis AI/ML prognostic prediction studies identified

Abbreviations: AI, artificial intelligence; ICU, intensive care unit; ML, machine learning.

Footnote: Exclusion counts are reported at the screening stage at which records were removed. The main reason column summarizes the dominant exclusion categories and is not intended as a mutually exclusive itemized list for every excluded record.

Supplementary Table S3. Study-level extraction summary of included studies

Complete study-level outcome mapping used for the main synthesis. The Ref. column preserves the original included-study extraction numbering used to identify the 75 studies in the supplementary material; it is not intended to indicate active citation numbering in the revised main manuscript. The corrected extraction table was reconciled against the 75 included full texts and yields the final descriptive counts used in the manuscript: AUROC/C-statistic extractable in 64/75 studies, external/temporal/prospective validation in 27/75, calibration in 38/75, decision-curve analysis or clinical-utility assessment in 37/75, and explainability/interpretable-output reporting in 64/75.

Ref.	Source	First author/year	Population / subgroup	Outcome predicted	Time horizon	Model family / best model	Validation / synthesis note	Calibration	DCA / utility	Explainability	Overall ROB	Reporting
1	PubMed	Jiang 2021	Adult ICU sepsis (ICU readmission)	In-hospital mortality	hospital stay	ML model(s) not clearly extracted / Not clearly extracted	Not clearly reported; AUROC/C-statistic reported	No/NR	No/NR	SHAP	High	Good
2	PubMed	Jiang 2023	Adult ICU sepsis	Clinically relevant outcome	variable	Boosting / XGBoost	External/clustered validation; AUROC: 0.731–0.828	No/NR	No/NR	SHAP	High	Good
3	PubMed	Zhi 2021	Adult ICU sepsis	In-hospital mortality	hospital stay	Random forest	External validation; AUROC: 0.495–0.776	Yes	No/NR	SHAP/feature importance	High	Limited
4	PubMed	Choi 2024	Adult ICU septic shock (elderly patients)	In-hospital mortality	hospital stay	Boosting; Random forest; SVM/classical ML; Deep learning / XGBoost	Cross-validation; Internal split/validation; AUROC/C-statistic reported	No/NR	No/NR	SHAP/feature importance	High	Good

Ref.	Source	First author/year	Population / subgroup	Outcome predicted	Time horizon	Model family / best model	Validation / synthesis note	Calibration	DCA / utility	Explainability	Overall ROB	Reporting
5	PubMed	Wernly 2021	Adult ICU sepsis	48-hour mortality / short-term mortality	48 hours	Deep learning / Logistic regression	Not clearly reported; AUROC: 0.720–0.880	No/NR	No/NR	Not reported	Some concerns	Good
6	PubMed	Bao 2023	Adult ICU sepsis (elderly patients; diabetes)	30-day mortality	30 days	Boosting; SVM/classical ML; Deep learning / XGBoost	Cross-validation; AUROC: 0.779–0.990	Yes	No/NR	Not reported	High	Good
7	PubMed	Ke 2022	Adult ICU septic shock (elderly patients; diabetes)	In-hospital mortality	hospital stay	Boosting; Random forest / XGBoost	Cross-validation; Internal split/validation; AUROC: 0.539–0.888	No/NR	No/NR	SHAP; feature importance	High	Good
8	PubMed	Gao 2024	Adult ICU sepsis	Mortality / poor prognosis	not clearly reported	Boosting; Random forest; SVM/classical ML / XGBoost	Cross-validation; AUROC: 0.940	No/NR	Yes	SHAP	High	Good
9	PubMed	Guo 2022	Adult ICU sepsis (sepsis-induced coagulopathy)	28-day mortality	28 days	Deep learning / Neural network/ANN	External validation; AUROC/C-statistic reported	Yes	Yes	SHAP/feature importance	High	Good
10	PubMed	Huang 2026	Adult ICU sepsis	ICU mortality	ICU stay	Survival ML	External validation; AUROC: Not clearly extracted	Yes	Yes	SHAP	High	Limited
11	PubMed	Jin 2026	Adult ICU septic shock (elderly patients)	In-hospital mortality	hospital stay	Deep learning; LLM/guideline-integrated / LSTM/RNN	Internal split/validation; AUROC: 0.740–0.829	Yes	No/NR	SHAP	High	Limited
12	PubMed	Kong 2020	Adult ICU sepsis	In-hospital mortality	hospital stay	Boosting; Random forest / GBM/GBDT	Not clearly reported; AUROC: Not clearly extracted	Yes	No/NR	Not reported	High	Good
13	PubMed	Li 2026	Adult ICU sepsis (cardiomyopathy/myocardial dysfunction)	Mortality	not clearly reported	Traditional/statistical + ML / Logistic regression	Cross-validation; Internal split/validation; AUROC: 0.770–0.940	Yes	Yes	SHAP	High	Moderate
14	PubMed	Luo 2025	Adult ICU sepsis (cancer patients)	Mortality / poor prognosis	not clearly reported	Boosting; Random forest	Cross-validation; AUROC: 0.760–0.800	Yes	Yes	SHAP	High	Moderate
15	PubMed	Ma 2023	Adult ICU sepsis (cancer patients)	In-hospital mortality	hospital stay	Traditional/statistical + ML / Logistic regression	Internal split/validation; AUROC: 0.722–0.837	Yes	Yes	nomogram/clinical interpretability	High	Moderate

Ref.	Source	First author/year	Population / subgroup	Outcome predicted	Time horizon	Model family / best model	Validation / synthesis note	Calibration	DCA / utility	Explainability	Overall ROB	Reporting
16	PubMed	Palmowski 2024	Adult ICU sepsis	30-day mortality	30 days	SVM/classical ML; Deep learning	External validation; Prospective cohort/evaluation; AUROC: 0.580–0.960	No/NR	No/NR	SHAP/feature importance	Some concerns	Good
17	PubMed	Pan 2023	Adult ICU sepsis	In-hospital mortality	hospital stay	SVM/classical ML	Cross-validation; Internal split/validation; AUROC/C-statistic reported	No/NR	Yes	SOFA-component interpretability	High	Good
18	PubMed	Pérez-Tomé 2024	Adult ICU sepsis	Mortality	not clearly reported	Random forest	Not clearly reported; AUROC: Not clearly extracted	No/NR	No/NR	Not reported	High	Limited
19	PubMed	Qi 2022	Adult ICU sepsis (diabetes)	In-hospital mortality	hospital stay	Boosting; Random forest / XGBoost	External validation; Internal split/validation; AUROC/C-statistic reported	No/NR	Yes	SHAP/feature importance	High	Good
20	PubMed	Rahman 2024	Adult ICU sepsis	30-day mortality	30 days	Boosting; Random forest / Logistic regression	Cross-validation; AUROC: 0.990	Yes	Yes	SHAP	High	Good
21	PubMed	Shen 2024	Adult ICU sepsis	28-day mortality	28 days	Boosting; SVM/classical ML / XGBoost	Cross-validation; AUROC: 0.703–0.821	No/NR	Yes	SHAP/feature importance	High	Good
22	PubMed	Shen 2025	Adult ICU sepsis (sepsis-associated delirium; elderly patients; cancer patients)	30-day mortality	30 days	Boosting; Random forest; Deep learning / GBM/GBDT	Cross-validation; Internal split/validation; AUROC: 0.816–0.874	Yes	Yes	SHAP; feature importance	High	Moderate
23	PubMed	Shi 2025	Adult ICU sepsis	Mortality	not clearly reported	ML model(s) not clearly extracted / Not clearly extracted	External validation; AUROC: Not clearly extracted	Yes	Yes	SHAP/feature importance	Some concerns	Good
24	PubMed	Su 2022	Adult ICU sepsis	30-day mortality	30 days	Deep learning / Logistic regression	Internal split/validation; AUROC: 0.619–0.873	No/NR	Yes	Not reported	High	Moderate
25	PubMed	Wang 2022	Adult ICU sepsis (sepsis-associated AKI; cancer patients; diabetes)	30-day mortality	30 days	Boosting; Random forest / XGBoost	Internal split/validation; AUROC: 0.485–0.943	Yes	No/NR	feature importance	Some concerns	Good
26	PubMed	Wang 2025	Adult ICU septic shock	In-hospital mortality	hospital stay	Boosting; SVM/classical	External validation; Internal	No/NR	No/NR	SHAP; feature importance	High	Good

Ref.	Source	First author/year	Population / subgroup	Outcome predicted	Time horizon	Model family / best model	Validation / synthesis note	Calibration	DCA / utility	Explainability	Overall ROB	Reporting
			(elderly patients; diabetes; ICU readmission)			ML; Deep learning / XGBoost	split/validation; AUROC: 0.551–0.880					
27	PubMed	Wei 2025	Adult ICU sepsis (urosepsis)	Mortality / poor prognosis	not clearly reported	Boosting; Random forest / XGBoost	Cross-validation; Internal split/validation; AUROC: 0.597–0.923	Yes	Yes	SHAP	High	Moderate
28	PubMed	Wu 2021	Adult ICU sepsis	In-hospital mortality	hospital stay	ML model(s) not clearly extracted / Not clearly extracted	External validation; AUROC: 0.781	Yes	No/NR	rule discovery	High	Limited
29	PubMed	Wu 2026	Adult ICU sepsis (sepsis-induced coagulopathy)	28-day mortality	28 days	Boosting / XGBoost	External validation; Internal split/validation; AUROC: 0.794–0.934	Yes	Yes	SHAP	Some concerns	Good
30	PubMed	Xu 2025	Adult ICU sepsis (sepsis-associated ARDS/acute respiratory failure)	In-hospital mortality	hospital stay	Boosting; Random forest; SVM/classical ML; Multimodal/imaging/omics	Internal split/validation; AUROC: 0.772–1.000	Yes	Yes	SHAP; feature importance	Some concerns	Good
31	PubMed	Yan 2024	Adult ICU septic shock (diabetes)	28-day mortality	28 days	Survival ML	Internal split/validation; AUROC: Not clearly extracted	Yes	Yes	Not reported	High	Limited
32	PubMed	Yong 2024	Adult ICU septic shock (elderly patients)	In-hospital mortality	hospital stay	Boosting; Random forest; SVM/classical ML; Deep learning; Survival ML	Internal split/validation; AUROC: Not clearly extracted	No/NR	No/NR	feature importance	High	Limited
33	PubMed	Yu 2024	Adult ICU sepsis	30-day mortality	30 days	Boosting / LightGBM	Not clearly reported; AUROC: 0.910–0.990	No/NR	No/NR	SHAP/feature importance	High	Moderate
34	PubMed	Zhang 2022	Adult ICU sepsis (elderly patients)	30-day mortality	30 days	Survival ML	Not clearly reported; AUROC: 0.731	Yes	No/NR	variable importance	High	Good
35	PubMed	Zhang 2024	Adult ICU sepsis	In-hospital mortality	hospital stay	Boosting / XGBoost	Not clearly reported; AUROC: 0.937–0.940	Yes	Yes	SHAP; feature importance	High	Good
36	PubMed	Zhang 2024	Adult ICU septic shock (sepsis-associated	In-hospital mortality	hospital stay	Traditional/statistical + ML /	Not clearly reported; AUROC: Not	Yes	No/NR	SHAP	High	Moderate

Ref.	Source	First author/year	Population / subgroup	Outcome predicted	Time horizon	Model family / best model	Validation / synthesis note	Calibration	DCA / utility	Explainability	Overall ROB	Reporting
			delirium; diabetes)			Logistic regression	clearly extracted					
37	PubMed	Zhang 2025	Adult ICU sepsis	Mortality, severity and/or ICU length of stay	variable	Boosting; Random forest / CatBoost	Internal split/validation; AUROC: 0.737–0.804	Yes	No/NR	SHAP	Some concerns	Good
38	PubMed	Zhao 2025	Adult ICU septic shock (sepsis-associated ARDS/acute respiratory failure; elderly patients; diabetes)	In-hospital mortality	hospital stay	Boosting; Random forest; SVM/classical ML; Deep learning; LLM/guideline-integrated; Multimodal/imaging/omics / GBM/GBDT	Cross-validation; Internal split/validation; AUROC: 0.678–0.852	No/NR	Yes	SHAP/feature importance	High	Limited
39	PubMed	Zhou 2024	Adult ICU sepsis (sepsis-induced coagulopathy)	28-day mortality	28 days	Boosting / XGBoost	External validation; Cross-validation; Internal split/validation; AUROC: 0.630–0.923	Yes	Yes	SHAP; LIME; feature importance	Some concerns	Good
40	PubMed	Zhou 2025	Adult ICU sepsis	Mortality	not clearly reported	Boosting; Random forest / LightGBM	Not clearly reported; AUROC: 0.760–0.790	No/NR	No/NR	SHAP; feature importance	High	Moderate
41	PubMed	Zhu 2025	Adult ICU sepsis (sepsis-associated ARDS/acute respiratory failure)	Mortality / poor prognosis	not clearly reported	Random forest; SVM/classical ML; Deep learning; Multimodal/imaging/omics	External validation; AUROC: 0.558–1.000	Yes	Yes	SHAP; feature importance	High	Good
42	PubMed	Zhuang 2023	Adult ICU sepsis	Mortality	not clearly reported	Boosting / XGBoost	External validation; Internal split/validation; AUROC: 0.830–0.870	Yes	Yes	SHAP	High	Good
43	PubMed	Huang 2023	Adult ICU septic shock	48-hour mortality / short-term mortality	48 hours	SVM/classical ML; Multimodal/imaging/omics	Cross-validation; Internal split/validation; AUROC: 0.820–0.960	No/NR	No/NR	Not reported	High	Moderate
44	PubMed	Wang 2022	Adult ICU sepsis	In-hospital mortality	hospital stay	ML model(s) not clearly extracted / Not clearly extracted	Internal split/validation; AUROC/C-statistic reported	Yes	No/NR	Not reported	High	Moderate
45	PubMed	Jiang 2025	Adult ICU sepsis (sepsis-associated	Mortality / poor prognosis	not clearly reported	ML model(s) not clearly extracted / Not	Not clearly reported;	Yes	Yes	SHAP/feature importance	Some concerns	Good

Ref.	Source	First author/year	Population / subgroup	Outcome predicted	Time horizon	Model family / best model	Validation / synthesis note	Calibration	DCA / utility	Explainability	Overall ROB	Reporting
			ARDS/acute respiratory failure)			clearly extracted	AUROC/C-statistic reported					
46	PubMed	Yin 2026	Adult ICU sepsis (sepsis-associated delirium; sepsis-associated AKI; elderly patients)	30-day mortality	30 days	Boosting; Random forest; SVM/classical ML; Deep learning / XGBoost	External validation; Internal split/validation; AUROC: 0.712–0.743	Yes	Yes	SHAP; feature importance	High	Moderate
47	PubMed	Li 2021	Adult ICU sepsis (elderly patients)	In-hospital mortality	hospital stay	Boosting; Random forest; SVM/classical ML / GBM/GBDT	Cross-validation; Internal split/validation; AUROC: 0.723–0.992	No/NR	No/NR	Not reported	High	Moderate
48	PubMed	Wang 2025	Adult ICU sepsis (sepsis-associated AKI; diabetes; hypoalbuminemia)	ICU mortality	ICU stay	Boosting; Random forest; SVM/classical ML / XGBoost	External validation; Cross-validation; Internal split/validation; AUROC: 0.453–0.924	Yes	Yes	SHAP; LIME	High	Moderate
49	PubMed	Seidlitz 2025	Adult ICU septic shock (cancer patients)	Mortality	not clearly reported	Random forest; Multimodal/imaging/omics	Prospective cohort/evaluation; AUROC: 0.590–0.920	Yes	No/NR	SHAP/feature importance	High	Limited
50	PubMed	Wang 2026	Adult ICU sepsis (diabetes)	28-day mortality	28 days	Boosting; Random forest; Survival ML / XGBoost	Not clearly reported; AUROC: 0.746–0.776	No/NR	No/NR	SHAP	Some concerns	Moderate
51	Scopus	Su 2021	Adult ICU septic shock	Mortality, severity and/or ICU length of stay	variable	Boosting; Random forest	Not clearly reported; AUROC: 0.500–0.840	No/NR	No/NR	feature importance	High	Moderate
52	Scopus	Xu 2026	Adult ICU sepsis (sepsis-associated ARDS/acute respiratory failure)	28-day mortality	28 days	Boosting; Random forest; Deep learning / XGBoost	External validation; AUROC: 0.605–0.823	No/NR	No/NR	SHAP; feature importance	Some concerns	Good
53	Scopus	Zheng 2023	Adult ICU septic shock	28-day mortality	28 days	Boosting / XGBoost	Cross-validation; AUROC/C-statistic reported	No/NR	Yes	SHAP/feature importance	Some concerns	Good
54	Scopus	Hu 2022	Adult ICU sepsis (ICU readmission)	In-hospital mortality	hospital stay	Random forest	Internal split/validation; AUROC: Not clearly extracted	No/NR	No/NR	SHAP; LIME	High	Moderate

Ref.	Source	First author/year	Population / subgroup	Outcome predicted	Time horizon	Model family / best model	Validation / synthesis note	Calibration	DCA / utility	Explainability	Overall ROB	Reporting
55	Scopus	Li 2024	Adult ICU sepsis	Mortality / poor prognosis	not clearly reported	Boosting; Random forest; SVM/classical ML / XGBoost	Not clearly reported; AUROC: 0.520–0.777	No/NR	No/NR	Not reported	Some concerns	Good
56	Scopus	Tang 2025	Adult ICU sepsis (cancer patients; diabetes)	ICU mortality	ICU stay	Boosting; Random forest; SVM/classical ML; Deep learning; Multimodal/imaging/omics / CatBoost	External validation; Cross-validation; Internal split/validation; AUROC: 0.673–0.945	Yes	Yes	SHAP; feature importance	Some concerns	Moderate
57	Scopus	Wang 2026	Adult ICU sepsis (sepsis-associated AKI; elderly patients)	In-hospital mortality	hospital stay	Boosting / XGBoost	Prospective cohort/evaluation; Internal split/validation; AUROC/C-statistic reported	No/NR	Yes	SHAP	Some concerns	Good
58	Scopus	Santos 2025	Adult ICU sepsis	In-hospital mortality	hospital stay	Boosting; Random forest; SVM/classical ML; Deep learning / XGBoost	External validation; AUROC: 0.507–0.840	No/NR	Yes	SHAP	High	Moderate
59	Scopus	Zhu 2025	Adult ICU sepsis (elderly patients)	Mortality	not clearly reported	Boosting / XGBoost	Internal split/validation; AUROC: 0.810–0.900	No/NR	Yes	SHAP; LIME	High	Moderate
60	Scopus	Li 2023	Adult ICU sepsis	In-hospital mortality	hospital stay	Boosting / XGBoost	Temporal validation; AUROC: 0.630–0.850	No/NR	No/NR	SHAP/feature importance	Some concerns	Good
61	Scopus	Diwan 2025	Adult ICU sepsis	Mortality	not clearly reported	Boosting; Random forest / XGBoost	Cross-validation; Internal split/validation; AUROC: 0.570–0.980	No/NR	No/NR	SHAP	Some concerns	Good
62	Scopus	Yao 2020	Adult ICU sepsis (sepsis-induced coagulopathy; cancer patients; diabetes)	In-hospital mortality	hospital stay	Boosting; Multimodal/imaging/omics / XGBoost	Not clearly reported; AUROC: 0.835	Yes	No/NR	Not reported	Some concerns	Moderate
63	Scopus	Li 2026	Adult ICU sepsis (sepsis-associated liver injury)	28-day mortality	28 days	Boosting / XGBoost	External validation; Internal split/validation; AUROC: 0.807–0.898	No/NR	No/NR	SHAP; LIME	Some concerns	Good
64	Scopus	Luo 2026	Adult ICU sepsis (sepsis-associated AKI)	In-hospital mortality	hospital stay	Boosting / GBM/GBDT	External validation; Internal split/validation;	Yes	Yes	SHAP	Some concerns	Good

Ref.	Source	First author/year	Population / subgroup	Outcome predicted	Time horizon	Model family / best model	Validation / synthesis note	Calibration	DCA / utility	Explainability	Overall ROB	Reporting
							AUROC: 0.731–0.847					
65	Scopus	Wang 2026	Adult ICU sepsis	28-day mortality	28 days	Traditional/statistical + ML / Logistic regression	External validation; Internal split/validation; AUROC: 0.771–0.787	Yes	Yes	SHAP/feature importance	Some concerns	Good
66	Scopus	Selcuk 2022	Adult ICU sepsis	Mortality	not clearly reported	Survival ML	Not clearly reported; AUROC/C-statistic not clearly extractable	Yes	Yes	SHAP/feature importance	High	Limited
67	Scopus	Zhuang 2025	Adult ICU sepsis (sepsis-associated AKI)	Mortality / poor prognosis	not clearly reported	Boosting; Survival ML / GBM/GBDT	External validation; Internal split/validation; AUROC: 0.694	No/NR	No/NR	SHAP	Some concerns	Good
68	Scopus	Yang 2023	Adult ICU sepsis (sepsis-associated AKI)	28-day mortality	28 days	Boosting; Survival ML / XGBoost	Cross-validation; AUROC: 0.873	Yes	Yes	SHAP	Some concerns	Good
69	Scopus	Ning 2023	Adult ICU septic shock	In-hospital mortality	hospital stay	Boosting; Deep learning; Survival ML / XGBoost	Not clearly reported; AUROC: Not clearly extracted	No/NR	No/NR	SHAP/feature importance	Some concerns	Moderate
70	Scopus	Zhang 2025	Adult ICU sepsis (sepsis-associated ARDS/acute respiratory failure)	ICU mortality	ICU stay	Boosting; Random forest; SVM/classical ML	External validation; Cross-validation; Internal split/validation; AUROC: 0.792–0.816	Yes	Yes	SHAP	Some concerns	Good
71	Scopus	Luo 2022	Adult ICU sepsis (sepsis-associated AKI)	In-hospital mortality	hospital stay	Boosting / XGBoost	External validation; Internal split/validation; AUROC: Not clearly extracted	No/NR	No/NR	SHAP	Some concerns	Good
72	Scopus	Sun 2025	Adult ICU sepsis	Mortality	not clearly reported	Deep learning / Neural network/ANN	Internal split/validation; AUROC: 0.698–0.787	Yes	Yes	SHAP	Some concerns	Good
73	Scopus	Guo 2023	Adult ICU sepsis	ICU mortality	ICU stay	Boosting / XGBoost	Internal split/validation; AUROC/C-statistic reported	No/NR	No/NR	SHAP	Some concerns	Moderate
74	Scopus	Amory 2025	Adult ICU sepsis	Mortality	not clearly reported	Boosting; Random forest / XGBoost	Internal split/validation; AUROC: 0.968	No/NR	Yes	SHAP/feature importance	Some concerns	Moderate

Ref.	Source	First author/year	Population / subgroup	Outcome predicted	Time horizon	Model family / best model	Validation / synthesis note	Calibration	DCA / utility	Explainability	Overall ROB	Reporting
75	Scopus	Hu 2022	Adult ICU sepsis	In-hospital mortality	hospital stay	Boosting / XGBoost	Not clearly reported; AUROC: 0.884	No/NR	No/NR	SHAP	Some concerns	Good

Abbreviations: AI, artificial intelligence; AKI, acute kidney injury; ANN, artificial neural network; ARDS, acute respiratory distress syndrome; AUROC, area under the receiver operating characteristic curve; DCA, decision-curve analysis; GBM/GBDT, gradient boosting machine/gradient boosting decision tree; ICU, intensive care unit; LIME, local interpretable model-agnostic explanations; ML, machine learning; NR, not reported; ROB, risk of bias; SALI, sepsis-associated liver injury; SHAP, Shapley additive explanations; SIC, sepsis-induced coagulopathy; SVM, support vector machine.

Footnote: Reference numbers follow the main manuscript. Validation status, calibration, DCA/utility and explainability were recorded as reported in each full text. "No/NR" denotes either absence of reporting or insufficient information for extraction. "Not clearly extracted" indicates that exact information was unavailable or not retained for the final quantitative count.

1. Jiang Z, Bo L, Xu Z, Song Y, Wang J, Wen P, et al. An explainable machine learning algorithm for risk factor analysis of in-hospital mortality in sepsis survivors with ICU readmission. Comput Methods Programs Biomed. 2021;204:106040. doi:10.1016/j.cmpb.2021.106040.
2. Jiang Z, Bo L, Wang L, Xie Y, Cao J, Yao Y, et al. Interpretable machine-learning model for real-time, clustered risk factor analysis of sepsis and septic death in critical care. Comput Methods Programs Biomed. 2023;241:107772. doi:10.1016/j.cmpb.2023.107772.
3. Zhi D, Zhang M, Lin J, Liu P, Wang Y, Duan M. Establishment and validation of the predictive model for the in-hospital death in patients with sepsis. Am J Infect Control. 2021. doi:10.1016/j.ajic.2021.07.010.
4. Choi JW, Yang M, Kim JW, Shin YM, Shin YG, Park S. Prognostic prediction of sepsis patient using transformer with skip connected token for tabular data. Artif Intell Med. 2024;149:102804. doi:10.1016/j.artmed.2024.102804.
5. Wernly B, Mamandipoor B, Baldia P, Jung C, Osmani V. Machine learning predicts mortality in septic patients using only routinely available ABG variables: A multi-centre evaluation. Int J Med Inform. 2021;145:104312. doi:10.1016/j.ijmedinf.2020.104312.
6. Bao C, Deng F, Zhao S. Machine-learning models for prediction of sepsis patients mortality. Med Intensiva. 2023;47:315-325. doi:10.1016/j.medine.2022.06.024.
7. Ke X, Wu Y, Liu S, Li Y, Chen Q. Interpretable machine learning to optimize early in-hospital mortality prediction for elderly patients with sepsis: A discovery study. Comput Math Methods Med. 2022;2022:4820464. doi:10.1155/2022/4820464.
8. Gao J, Lu Y, Ashrafi N, Domingo I, Alaei K, Pishgar M. Prediction of sepsis mortality in ICU patients using machine learning methods. BMC Med Inform Decis Mak. 2024;24:228. doi:10.1186/s12911-024-02630-z.
9. Guo F, Zhu X, Wu Z, Zhu L, Wu J, Zhang F. Clinical applications of machine learning in the survival prediction and classification of sepsis: coagulation and heparin usage matter. J Transl Med. 2022;20:265. doi:10.1186/s12967-022-03469-6.
10. Huang S, Liu L, Wang C, Li X, Liu Y, Ma X, et al. A machine learning-based prediction model for poor prognosis in sepsis using lymphocyte count: a national, multicenter prospective cohort. Sci Rep. 2026;16:3816. doi:10.1038/s41598-025-33980-x.
11. Jin H, Ashrafi N, Alaei K, Pishgar E, Placencia G, Pishgar M. A novel multi-task teacher-student architecture with self-supervised pretraining for 48-hour vasoactive-inotropic trend analysis in sepsis mortality prediction. IEEE J Biomed Health Inform. 2026;30(3):1986-1999. doi:10.1109/JBHI.2025.3609667.
12. Kong G, Lin K, Hu Y. Using machine learning methods to predict in-hospital mortality of sepsis patients in the ICU. BMC Med Inform Decis Mak. 2020;20:251. doi:10.1186/s12911-020-01271-2.
13. Li X, Cheng H, Ainiwaer D, Yang L, Duan Q, Wang Z, et al. Enhancing prognostic accuracy in sepsis-induced cardiomyopathy: a machine learning approach. Eur J Med Res. 2026;31:46. doi:10.1186/s40001-025-03354-0.
14. Luo X, Zhai Y, Luo Y, Xie J, Wu H, Xu X, et al. Development and validation of an explainable machine learning model for predicting prognosis in sepsis patients with a history of cancer who were admitted to the intensive care unit. J Int Med Res. 2025;53(8):3000605251362991. doi:10.1177/03000605251362991.
15. Ma CY, Sun GR, Yang XW, Yang S. A clinically applicable prediction model for the risk of in-hospital mortality in solid cancer patients admitted to intensive care units with sepsis. J Cancer Res Clin Oncol. 2023;149:7175-7185. doi:10.1007/s00432-023-04661-x.

16. Palmowski L, Nowak H, Witowski A, Koos B, Wolf A, Weber M, et al. Assessing SOFA score trajectories in sepsis using machine learning: a pragmatic approach to improve the accuracy of mortality prediction. PLoS One. 2024;19(3):e0300739. doi:10.1371/journal.pone.0300739.

17. Pan X, Xie J, Zhang L, Wang X, Zhang S, Zhuang Y, et al. Evaluate prognostic accuracy of SOFA component score for mortality among adults with sepsis by machine learning. BMC Infect Dis. 2023;23:197. doi:10.1186/s12879-023-08045-x.

18. Pérez-Tomé JC, Parrón-Carreño T, Castaño-Fernández AB, Nievas-Soriano BJ, Castro-Luna G. Sepsis mortality prediction with machine learning techniques. Med Intensiva. 2024. PMID:38876921.

19. Qi J, Lei J, Li N, Huang D, Liu H, Zhou K, et al. Machine learning models to predict in-hospital mortality in septic patients with diabetes. Front Endocrinol. 2022;13:1034251. doi:10.3389/fendo.2022.1034251.

20. Rahman MS, Islam KR, Prithula J, Kumar J. Machine learning-based prognostic model for 30-day mortality prediction in Sepsis-3. BMC Med Inform Decis Mak. 2024;24:249. doi:10.1186/s12911-024-02655-4.

21. Shen Y, Liao X, Sun X, Yang P, Zhang J, Yu Y, et al. Interpretable machine learning-based prediction of 28-day mortality in ICU patients with sepsis. Front Cell Infect Microbiol. 2024;14:1500326. doi:10.3389/fcimb.2024.1500326.

22. Shen Y, Liao X, Sun X, Yang P, Zhang J, Yu Y, et al. Machine learning approach for the prediction of 30-day mortality in patients with sepsis-associated complications. PLoS One. 2025;20(4):e0319519. doi:10.1371/journal.pone.0319519.

23. Shi S, Zhang L, Zhang S, Shi J, Hong D, Wu S, et al. Developing a rapid screening tool for high-risk ICU patients of sepsis: integrating electronic medical records with machine learning methods for mortality prediction. J Transl Med. 2025;23:97. doi:10.1186/s12967-025-06102-4.

24. Su Y, Guo C, Zhou S, Li C, Ding N. Early predicting 30-day mortality in sepsis in MIMIC-III by an artificial neural networks model. Eur J Med Res. 2022;27:294. doi:10.1186/s40001-022-00925-3.

25. Wang H, Li Y, Naidech A, Luo Y. Comparison between machine learning methods for mortality prediction for sepsis patients with different clinical phenotypes. BMC Med Inform Decis Mak. 2022;22:156. doi:10.1186/s12911-022-01871-0.

26. Wang Y, Gao Z, Zhang Y, Lu Z, Sun F. Early sepsis mortality prediction model based on interpretable machine learning approach: development and validation study. Intern Emerg Med. 2025;20:909-918. doi:10.1007/s11739-024-03732-2.

27. Wei Y, Xu W, Yang S, Zhang C, Wang J, Wan X, et al. Significant adverse prognostic events in patients with urosepsis: a machine learning-based model development and validation study. Front Cell Infect Microbiol. 2025;15:1623109. doi:10.3389/fcimb.2025.1623109.

28. Wu Y, Huang S, Chang X. Understanding the complexity of sepsis mortality prediction via rule discovery and analysis: a pilot study. BMC Med Inform Decis Mak. 2021;21:334. doi:10.1186/s12911-021-01690-9.

29. Wu J, Zhang X, Liang C, Wang B, Ruan X, Dong Y, et al. Explainable machine learning model for predicting short-term outcomes in sepsis-induced coagulopathy. BMC Med Inform Decis Mak. 2026;26:67. doi:10.1186/s12911-026-03363-x.

30. Xu Z, Ni X, Song Y, Chen Q, Xu T, Sun C, et al. Predicting mortality and risk factors of sepsis-related ARDS using machine learning models. Sci Rep. 2025;15:1467366. doi:10.1038/s41598-025-96501-w.

31. Yan D, Zhang Y, Liu S, Chen J, He Y, Wu X, et al. Construction and evaluation of short-term and long-term mortality risk prediction model for patients with sepsis. Zhong Nan Da Xue Xue Bao Yi Xue Ban. 2024;49(2):256-265. doi:10.11817/j.issn.1672-7347.2024.230390.

32. Yong X, Zhenzhou L. Deep learning-based prediction of in-hospital mortality for sepsis. Sci Rep. 2024. doi:10.1038/s41598-023-49890-9.

33. Yu Z, Ashrafi N, Li H, Alaei K, Pishgar M. Prediction of 30-day mortality for ICU patients with Sepsis-3. BMC Med Inform Decis Mak. 2024;24:247. doi:10.1186/s12911-024-02629-6.

34. Zhang L, Huang T, Xu F, Li S, Zheng S, Lyu J, et al. Prediction of prognosis in elderly patients with sepsis based on machine learning. BMC Emerg Med. 2022;22:26. doi:10.1186/s12873-022-00582-z.

35. Zhang G, Shao F, Yuan W, Wu J, Qi X, Gao J, et al. Predicting sepsis in-hospital mortality with machine learning: a multi-center study using clinical and inflammatory biomarkers. Eur J Med Res. 2024;29:156. doi:10.1186/s40001-024-01756-0.

36. Zhang L, Li X, Huang J, Yang Y, Peng H, Yang L, et al. Predictive model of risk factors for 28-day mortality in patients with sepsis or sepsis-associated delirium. Sci Rep. 2024;14:18562. doi:10.1038/s41598-024-69332-4.

37. Zhang S, Zhou H, Zhou M, Li H, Wu J, Li R, et al. Harness machine learning for multiple prognoses prediction in sepsis patients: evidence from the MIMIC database. BMC Med Inform Decis Mak. 2025;25:241. doi:10.1186/s12911-025-02976-y.

38. Zhao Z, An B, Zhang T, Zhu R, Fan Z, Wang G. Integrating clinical guidelines with large language models for improved sepsis mortality prediction. Health Informatics Journal. 2025;31(4):1-18. doi:10.1177/14604582251387649.

39. Zhou S, Lu Z, Liu Y, Wang M, Zhou W, Cui X, et al. Interpretable machine learning model for early prediction of 28-day mortality in ICU patients with sepsis-induced coagulopathy: development and validation. Eur J Med Res. 2024;29:14. doi:10.1186/s40001-023-01593-7.

40. Zhou P, Qin Y, Xu P, Li J, Liu Q, He R, et al. Improving sepsis mortality prediction with machine learning: a comparative study of advanced classification algorithms. Adv Clin Exp Med. 2025. doi:10.17219/acem/194660.

41. Zhu L, Chen Z, Zhang H, Chen H, Liu L, Yu W, et al. Explainable AI unravels sepsis heterogeneity via coagulation-inflammation profiles for prognosis and stratification. Nat Commun. 2025;16:10396. doi:10.1038/s41467-025-65365-z.

42. Zhuang J, Huang H, Jiang S, Liang J, Liu Y, Yu X. A generalizable and interpretable model for mortality risk stratification of sepsis patients in intensive care units. BMC Med Inform Decis Mak. 2023;23:229. doi:10.1186/s12911-023-02279-0.

43. Huang M, Atreya MR, Holder A, Kamaleswaran R. A machine learning model derived from analysis of time-course gene-expression datasets reveals temporal gene signatures associated with sepsis mortality. Shock. 2023. doi:10.1097/SHK.0000000000002226.

44. Wang Y, Zhang H, Chen J, Liu Y, Li W, et al. A prediction model for 30-day mortality of sepsis patients based on machine learning. Medicine. 2022;101:e30578. doi:10.1097/MD.0000000000030578.

45. Jiang L, Yu C, Xie C, Zheng Y, Xia Z, et al. Enhancing early mortality prediction for sepsis-associated acute respiratory distress syndrome patients via optimized machine learning algorithm. Int J Surg. 2025. doi:10.1097/JS9.0000000000002741.

46. Yin J, Pan X, Chen D, Zhang J, Jin G. Machine-learning model for 30-day mortality in sepsis-associated delirium patients: A retrospective MIMIC-IV cohort study. Medicine. 2026. doi:10.1097/MD.0000000000045440.

47. Li K, Shi Q, Liu S, Xie Y, Liu J. Predicting in-hospital mortality in ICU patients with sepsis using gradient boosting decision tree. Medicine. 2021;100:e25813. doi:10.1097/MD.0000000000025813.

48. Wang Z, Huang B, Peng H, Li J, Wu X, He Z, et al. Prediction of the mortality rate in the intensive care unit for patients with combined hypoalbuminemia based on machine learning. Medicine. 2025;104:e43610. doi:10.1097/MD.0000000000043610.

49. Seidlitz S, Hölzl K, von Garrel A, Arbab M, Dierks J, Guttmann J, et al. AI-powered skin spectral imaging enables instant sepsis diagnosis and outcome prediction in critically ill patients. Sci Adv. 2025;11:eadw1968. doi:10.1126/sciadv.adw1968.

50. Wang F, Jin X, Liu X, Yan Q, Jiang L, Chen D, et al. Simultaneous assessment of stress hyperglycemia ratio and glucose variability to predict all-cause mortality in sepsis patients across different glucose metabolic states. Int J Surg. 2026. doi:10.1097/JS9.0000000000003525.

51. Su L, Xu Z, Chang F, Ma Y, Liu S, Jiang H, et al. Early prediction of mortality, severity and ICU length of stay in Sepsis-3 ICU patients. Front Med. 2021;8:664966. doi:10.3389/fmed.2021.664966.

52. Xu Y, Lei T, Yang Z, Guo H, Zhu L, Wang J, et al. Development and external validation of a machine learning model for 28-day mortality in sepsis with acute respiratory failure in ICU. J Intensive Med. 2026;6:175-184. doi:10.1016/j.jointm.2025.10.010.

53. Zheng F, Wang L, Pang Y, Chen Z, Lu Y, Yang Y, et al. ShockSurv: machine learning model to predict 28-day mortality for septic shock patients in ICU. Biomed Signal Process Control. 2023;86:105146. doi:10.1016/j.bspc.2023.105146.

54. Hu C, Li L, Li Y, Wang F, Hu B, Peng Z. Explainable machine learning model for in-hospital mortality in septic patients requiring ICU readmission. Infect Dis Ther. 2022;11:1869-1885. doi:10.1007/s40121-022-00671-3.

55. Li C, Wang L, Li K, Deng H, Wang Y, Chang L, et al. Machine-learning-enabled prognostic models for sepsis. Intell Med. 2024. doi:10.1016/j.ibmed.2024.100167.
56. Tang H, Hao H, Han Y. Personalized ICU mortality assessment in sepsis combined with lung cancer. Front Oncol. 2025;15:1661212. doi:10.3389/fonc.2025.1661212.
57. Wang JZ, Zhang N, Ma RR, Yang M, Chen YG, Zhou WJ. An interpretable machine-learning model for predicting in-hospital mortality in patients with sepsis-associated acute kidney injury. Front Med. 2026;13:1756831. doi:10.3389/fmed.2026.1756831.
58. Santos C, Ramos Garzón JX, Pertuz SD, Fajardo CA. Significant clinical factors for mortality prediction in ICU sepsis patients: a machine learning approach. Smart Health. 2025;37:100613. doi:10.1016/j.smhl.2025.100613.
59. Zhu XY, Jiang ZM, Li X, Lv ZW, Tian JW, Su FF. Interpretive machine learning predicts short-term mortality risk in elderly sepsis patients. Front Physiol. 2025;16:1549138. doi:10.3389/fphys.2025.1549138.
60. Li S, Dou R, Song X, Lui KY, Xu J, Guo Z, et al. Developing an interpretable machine learning model to predict in-hospital mortality in sepsis patients: a retrospective temporal validation study. J Clin Med. 2023;12:915. doi:10.3390/jcm12030915.
61. Diwan S, Gandhi V, Kayal EB, Khanna P, Mehndiratta A. Explainable machine learning models for mortality prediction in patients with sepsis in tertiary care hospital ICU in low- to middle-income countries. Intensive Care Med Exp. 2025;13:56. doi:10.1186/s40635-025-00765-5.
62. Yao RQ, Jin X, Wang GW, Yu Y, Wu GS, Zhu YB, et al. A machine learning-based prediction of hospital mortality in patients with postoperative sepsis. Front Med. 2020;7:445. doi:10.3389/fmed.2020.00445.
63. Li Y, Fan J, Alaei K, Pishgar M. Development of machine learning models to predict 28-day mortality in patients with sepsis-associated liver injury. Biomedinformatics. 2026;6:4. doi:10.3390/biomedinformatics6010004.
64. Luo S, Lai J, Mo L, Shen X, Fang R. Prediction of hospital mortality in sepsis-associated acute kidney injury using a machine-learning approach: a multicenter study using SHAP interpretability analysis. Clin Kidney J. 2026;19:sfaf372. doi:10.1093/ckj/sfaf372.
65. Wang Z, Xiao X, Li S, He J, Li Y, Huang F, et al. Development and validation of a 28-day mortality prediction model for patients with sepsis complicated by autoimmune diseases using two machine learning methods. J Inflamm Res. 2026;19:555953. doi:10.2147/JIR.S555953.
66. Selcuk M, Koc O, Kestel AS. The prediction power of machine learning on estimating the sepsis mortality in the intensive care unit. Inform Med Unlocked. 2022;28:100861. doi:10.1016/j.imu.2022.100861.
67. Zhuang C, Hu R, Li K, Liu Z, Bai S, Zhang S, et al. Machine learning prediction models for mortality risk in sepsis-associated acute kidney injury evaluating early versus late continuous renal replacement therapy. Front Med. 2025;12:1483710. doi:10.3389/fmed.2024.1483710.
68. Yang J, Peng H, Luo Y, Zhu T, Xie L. Explainable ensemble machine learning model for prediction of 28-day mortality risk in patients with sepsis-associated acute kidney injury. Front Med. 2023;10:1165129. doi:10.3389/fmed.2023.1165129.
69. Ning YL, Sun C, Xu XH, Li L, Ke YJ, Mai Y, et al. Tendency of dynamic vasoactive and inotropic medications data as a robust predictor of mortality in patients with septic shock. Front Cardiovasc Med. 2023;10:1126888. doi:10.3389/fcvm.2023.1126888.
70. Zhang P, Yuan S, Zhang S, Yuan Z, Ye Z, Lv L, et al. Under the background of the new global definition of ARDS: an interpretable machine learning approach for predicting 28-day ICU mortality in patients with sepsis complicated by ARDS. Front Physiol. 2025;16:1617196. doi:10.3389/fphys.2025.1617196.
71. Luo XQ, Yan P, Duan SB, Kang YX, Deng YH, Liu Q, et al. Development and validation of machine learning models for real-time mortality prediction in critically ill patients with sepsis-associated acute kidney injury. Front Med. 2022;9:853102. doi:10.3389/fmed.2022.853102.
72. Sun W, Zhang L, Mou D, Zhao B, Che Z, Li Y, et al. Interpretable machine learning model for early mortality prediction in septic patients using routine post-diagnosis clinical data: a multicenter study. J Inflamm Res. 2025;18:553042. doi:10.2147/JIR.S553042.
73. Guo J, Cheng H, Wang Z, Qiao M, Li J, Lyu J. Factor analysis based on SHapley Additive exPlanations for sepsis-associated encephalopathy in ICU mortality prediction using XGBoost. Front Neurol. 2023;14:1290117. doi:10.3389/fneur.2023.1290117.
74. Amory IA, Rashidi Khazaee P, Yousefi S. Improving sepsis mortality prediction with machine learning using full region synthetic sampling approach. Health Sci Rep. 2025;8:e71556. doi:10.1002/hsr2.71556.

75. Hu C, Li L, Huang W, Wu T, Xu Q, Liu J, et al. Interpretable machine learning for early prediction of prognosis in sepsis: a discovery and validation study. Infect Dis Ther. 2022;11:1117-1132. doi:10.1007/s40121-022-00628-6.

Supplementary Table S4. Study-level PROBAST/PROBAST+AI assessment

Ref.	First author/year	Participants	Predictors	Outcome	Analysis	AI-specific concerns	Applicability	Overall risk of bias
19	Jiang 2021	SC	SC	SC	H	H	SC	H
20	Jiang 2023	SC	SC	SC	H	H	SC	H
21	Zhi 2021	SC	SC	SC	H	H	SC	H
22	Choi 2024	SC	SC	SC	H	H	SC	H
23	Wernly 2021	SC	SC	SC	SC	SC	SC	SC
24	Bao 2023	SC	SC	SC	H	H	SC	H
25	Ke 2022	SC	SC	SC	H	H	SC	H
26	Gao 2024	SC	SC	SC	H	H	SC	H
27	Guo 2022	SC	SC	SC	H	H	SC	H
28	Huang Y 2026	SC	SC	SC	H	H	SC	H
29	Jin 2026	SC	SC	SC	H	H	SC	H
30	Kong 2020	SC	SC	SC	H	H	SC	H
31	Li 2026	SC	SC	SC	H	H	SC	H
32	Luo 2025	SC	SC	SC	H	H	SC	H
33	Ma 2023	SC	SC	SC	H	H	SC	H
34	Palmowski 2024	SC	SC	SC	SC	SC	SC	SC
35	Pan 2023	SC	SC	SC	H	H	SC	H
36	Pérez-Tomé 2024	SC	SC	SC	H	H	SC	H
37	Qi 2022	SC	SC	SC	H	H	SC	H
38	Rahman 2024	SC	SC	SC	H	H	SC	H
39	Shen 2024	SC	SC	SC	H	H	SC	H
40	Shen 2025	SC	SC	SC	H	H	SC	H
41	Shi 2025	SC	SC	SC	SC	SC	SC	SC
42	Su 2022	SC	SC	SC	H	H	SC	H
43	Wang Y 2022	SC	SC	SC	SC	SC	SC	SC
44	Wang Y 2025	SC	SC	SC	H	H	SC	H
45	Wei 2025	SC	SC	SC	H	H	SC	H
46	Wu 2021	SC	SC	SC	H	H	SC	H
47	Wu 2026	SC	SC	SC	SC	SC	SC	SC
48	Xu Y 2025	SC	SC	SC	SC	SC	SC	SC
49	Yan 2024	SC	SC	SC	H	H	SC	H
50	Yong 2024	SC	SC	SC	H	H	SC	H

Ref.	First author/year	Participants	Predictors	Outcome	Analysis	AI-specific concerns	Applicability	Overall risk of bias
51	Yu 2024	SC	SC	SC	H	H	SC	H
52	Zhang Z 2022	SC	SC	SC	H	H	SC	H
53	Zhang Y 2024	SC	SC	SC	H	H	SC	H
54	Zhang Y 2024	SC	SC	SC	H	H	SC	H
55	Zhang Y 2025	SC	SC	SC	SC	SC	SC	SC
56	Zhao 2025	SC	SC	SC	H	H	SC	H
57	Zhou 2024	SC	SC	SC	SC	SC	SC	SC
58	Zhou 2025	SC	SC	SC	H	H	SC	H
59	Zhu 2025	SC	SC	SC	H	H	SC	H
60	Zhuang 2023	SC	SC	SC	H	H	SC	H
61	Huang M 2023	SC	SC	SC	H	H	SC	H
62	Wang Y 2022	SC	SC	SC	H	H	SC	H
63	Jiang L 2025	SC	SC	SC	SC	SC	SC	SC
64	Yin 2026	SC	SC	SC	H	H	SC	H
65	Li X 2021	SC	SC	SC	H	H	SC	H
66	Wang X 2025	SC	SC	SC	H	H	SC	H
67	Seidlitz 2025	SC	SC	SC	H	H	SC	H
68	Wang Y 2026	SC	SC	SC	SC	SC	SC	SC
69	Su 2021	SC	SC	SC	H	H	SC	H
70	Xu 2026	SC	SC	SC	SC	SC	SC	SC
71	Zheng 2023	SC	SC	SC	SC	SC	SC	SC
72	Hu 2022	SC	SC	SC	H	H	SC	H
73	Li Y 2024	SC	SC	SC	SC	SC	SC	SC
74	Tang 2025	SC	SC	SC	SC	SC	SC	SC
75	Wang Y 2026	SC	SC	SC	SC	SC	SC	SC
76	Santos 2025	SC	SC	SC	H	H	SC	H
77	Zhu Y 2025	SC	SC	SC	H	H	SC	H
78	Li X 2023	SC	SC	SC	SC	SC	SC	SC
79	Diwan 2025	SC	SC	SC	SC	SC	SC	SC
80	Yao 2020	SC	SC	SC	SC	SC	SC	SC
81	Li 2026	SC	SC	SC	SC	SC	SC	SC
82	Luo 2026	SC	SC	SC	SC	SC	SC	SC
83	Wang Y 2026	SC	SC	SC	SC	SC	SC	SC
84	Selcuk 2022	SC	SC	SC	H	H	SC	H
85	Zhuang 2025	SC	SC	SC	SC	SC	SC	SC
86	Yang 2023	SC	SC	SC	SC	SC	SC	SC
87	Ning 2023	SC	SC	SC	SC	SC	SC	SC

Ref.	First author/year	Participants	Predictors	Outcome	Analysis	AI-specific concerns	Applicability	Overall risk of bias
88	Zhang Y 2025	SC	SC	SC	SC	SC	SC	SC
89	Luo 2022	SC	SC	SC	SC	SC	SC	SC
90	Sun 2025	SC	SC	SC	SC	SC	SC	SC
91	Guo Y 2023	SC	SC	SC	SC	SC	SC	SC
92	Amory 2025	SC	SC	SC	SC	SC	SC	SC
93	Hu C 2022	SC	SC	SC	SC	SC	SC	SC

Abbreviations: AI, artificial intelligence; H, high risk of bias; ICU, intensive care unit; PROBAST, Prediction model Risk Of Bias ASsessment Tool; ROB, risk of bias; SC, some concerns.

Footnote: Judgments were conservative and primarily driven by analysis-domain and AI-specific concerns, including validation strategy, overfitting, leakage, missing-data handling, hyperparameter tuning, reproducibility and external validation. Applicability was interpreted in relation to adult ICU sepsis prognostic prediction.

Supplementary Table S5. TRIPOD/TRIPOD+AI reporting assessment

Ref.	First author/year	Data source / participants	Predictors / feature timing	Model development / validation	Performance, calibration and utility	Explainability / availability	Overall reporting
19	Jiang 2021	A	P	A	P	A	Good
20	Jiang 2023	A	P	A	P	A	Good
21	Zhi 2021	P	P	P	P	NR	Limited
22	Choi 2024	A	P	A	P	P	Good
23	Wernly 2021	A	A	A	P	P	Good
24	Bao 2023	A	P	A	P	P	Good
25	Ke 2022	A	P	A	P	A	Good
26	Gao 2024	A	P	A	P	P	Good
27	Guo 2022	A	P	A	P	P	Good
28	Huang Y 2026	P	P	P	P	NR	Limited
29	Jin 2026	P	P	P	P	NR	Limited
30	Kong 2020	A	A	A	A	P	Good
31	Li 2026	P	P	P	P	P	Moderate
32	Luo 2025	P	P	P	P	A	Moderate
33	Ma 2023	P	P	P	P	P	Moderate
34	Palmowski 2024	A	A	A	A	P	Good
35	Pan 2023	A	P	A	P	P	Good
36	Pérez-Tomé 2024	P	P	P	P	NR	Limited
37	Qi 2022	A	P	A	P	P	Good
38	Rahman 2024	A	P	A	P	P	Good
39	Shen 2024	A	P	A	P	A	Good
40	Shen 2025	P	P	P	P	P	Moderate
41	Shi 2025	A	A	A	P	P	Good
42	Su 2022	P	P	P	P	P	Moderate

Ref.	First author/year	Data source / participants	Predictors / feature timing	Model development / validation	Performance, calibration and utility	Explainability / availability	Overall reporting
43	Wang Y 2022	A	P	A	P	A	Good
44	Wang Y 2025	A	P	A	P	A	Good
45	Wei 2025	P	P	P	P	P	Moderate
46	Wu 2021	P	P	P	NR	P	Limited
47	Wu 2026	A	A	A	A	A	Good
48	Xu Y 2025	A	P	A	P	A	Good
49	Yan 2024	P	P	P	P	NR	Limited
50	Yong 2024	P	P	P	P	NR	Limited
51	Yu 2024	P	P	P	P	P	Moderate
52	Zhang Z 2022	A	P	A	P	P	Good
53	Zhang Y 2024	A	A	A	P	P	Good
54	Zhang Y 2024	P	P	P	P	P	Moderate
55	Zhang Y 2025	A	P	A	P	A	Good
56	Zhao 2025	P	P	P	NR	P	Limited
57	Zhou 2024	A	A	A	A	A	Good
58	Zhou 2025	P	P	P	P	P	Moderate
59	Zhu 2025	A	P	A	P	A	Good
60	Zhuang 2023	A	P	A	P	A	Good
61	Huang M 2023	P	P	P	P	A	Moderate
62	Wang Y 2022	P	P	P	P	P	Moderate
63	Jiang L 2025	A	A	A	A	A	Good
64	Yin 2026	P	P	P	P	P	Moderate
65	Li X 2021	P	P	P	P	P	Moderate
66	Wang X 2025	P	P	P	P	P	Moderate
67	Seidlitz 2025	P	P	P	NR	P	Limited
68	Wang Y 2026	P	P	P	P	P	Moderate
69	Su 2021	P	P	P	P	P	Moderate
70	Xu 2026	A	A	A	A	P	Good
71	Zheng 2023	A	A	A	A	P	Good
72	Hu 2022	P	P	P	P	A	Moderate
73	Li Y 2024	A	P	A	P	P	Good
74	Tang 2025	P	P	P	P	P	Moderate
75	Wang Y 2026	A	A	A	A	A	Good
76	Santos 2025	P	P	P	P	P	Moderate
77	Zhu Y 2025	P	P	P	P	A	Moderate
78	Li X 2023	A	A	A	A	A	Good

Ref.	First author/year	Data source / participants	Predictors / feature timing	Model development / validation	Performance, calibration and utility	Explainability / availability	Overall reporting
79	Diwan 2025	A	P	A	P	A	Good
80	Yao 2020	P	P	P	P	P	Moderate
81	Li 2026	A	A	A	A	P	Good
82	Luo 2026	A	A	A	A	P	Good
83	Wang Y 2026	A	A	A	A	A	Good
84	Selcuk 2022	P	P	P	NR	NR	Limited
85	Zhuang 2025	A	P	A	P	A	Good
86	Yang 2023	A	A	A	A	A	Good
87	Ning 2023	P	P	P	P	P	Moderate
88	Zhang Y 2025	A	P	A	P	A	Good
89	Luo 2022	A	A	A	P	P	Good
90	Sun 2025	A	A	A	A	P	Good
91	Guo Y 2023	P	P	P	P	A	Moderate
92	Amory 2025	P	P	P	P	P	Moderate
93	Hu C 2022	A	A	A	A	A	Good

Abbreviations: A, adequate; AI, artificial intelligence; NR, not reported or poor; P, partial; PROBAST, Prediction model Risk Of Bias ASsessment Tool; TRIPOD, Transparent Reporting of a multivariable prediction model for Individual Prognosis Or Diagnosis.

Footnote: TRIPOD/TRIPOD+AI was used to assess reporting completeness rather than methodological validity. Reporting judgments were interpreted alongside the PROBAST/PROBAST+AI risk-of-bias assessment.

Supplementary Table S6. Quantitative synthesis feasibility matrix

Formal meta-analysis was not performed as the primary synthesis because no outcome cluster met predefined criteria for sufficiently homogeneous population, time horizon, validation setting and uncertainty reporting.

Candidate outcome cluster	Studies with reported AUROC/C-statistic	Main barriers to pooling	Decision
ICU mortality	Several studies	Heterogeneous populations, prediction time zero, model families and validation strategies	Narrative synthesis only
In-hospital mortality	Frequently reported	Database overlap, variable populations, inconsistent confidence intervals, internal/external validation mixed	Narrative synthesis only
7-day / 96-hour mortality	Few studies	Different time horizons and clinical interpretation	Narrative synthesis only
28-day mortality	Several studies	Different subgroups including SIC, septic shock, liver injury, S-AKI and autoimmune disease-associated sepsis; inconsistent CI/SE reporting; high heterogeneity	No primary meta-analysis
30-day mortality	Several studies	Fewer homogeneous studies; subgroup-specific populations; variable validation	Narrative synthesis only
Long-term mortality	Limited studies	Different horizons, survival models and binary classifiers not directly comparable	Narrative synthesis only
Non-mortality outcomes	Heterogeneous	Different definitions, timing and clinical meaning	Narrative synthesis only

Candidate outcome cluster	Studies with reported AUROC/C-statistic	Main barriers to pooling	Decision
AI/ML versus conventional scores	17 directly extractable paired comparisons from 9 studies	Paired ΔAUROC uncertainty, ROC covariance and DeLong CI rarely reported; one comparison favored a conventional score; exact values were retained only when directly extractable from full text or uploaded supplementary materials	Descriptive comparative synthesis only

Abbreviations: AUROC, area under the receiver operating characteristic curve; CI, confidence interval; DCA, decision-curve analysis; ICU, intensive care unit; ML, machine learning.

Footnote: The matrix summarizes whether formal quantitative pooling was justified. Meta-analysis was not performed because of heterogeneity in population, time zero, prediction horizon, validation setting and uncertainty reporting.

Supplementary Table S7. Directly extractable AI/ML versus conventional severity-score comparisons

Only within-study comparisons with exact numeric AUROC/C-statistic values directly extractable from the full text or uploaded supplementary materials were retained. ΔAUROC = AI/ML AUROC minus the AUROC of the best-performing conventional comparator within the same outcome and validation/data setting.

Variable	Value
Included studies	9
Directly extractable paired comparisons	17
Median paired ΔAUROC	+0.108
IQR paired ΔAUROC	+0.082 to +0.148
Range paired ΔAUROC	−0.013 to +0.203
Interpretation	Usually, but not uniformly, higher discrimination for AI/ML; descriptive only, not formal superiority or non-inferiority.

Ref.	Study	Outcome/comparison	AI/ML model	Validation/data setting	AI AUROC	Best conventional comparator	Comparator AUROC	ΔAUROC	Verification note
22	Choi 2024	Mortality	skip-connected token Transformer/proposed model	internal/cross-validation	0.8047	APACHE II	0.6019	0.2028	full-text lines: proposed model AUROC 0.8047; SOFA 0.5687; APACHE II 0.6019
23	Wernly 2021	96-h mortality MIMIC-III	LSTM	single-centre MIMIC-III	0.85	SOFA	0.76	0.09	full text: LSTM 0.85; SOFA 0.76
23	Wernly 2021	96-h mortality eICU	LSTM	multi-centre eICU	0.88	SOFA	0.72	0.16	full text: LSTM 0.88; SOFA 0.72
55	Zhang 2025	Chronic critical illness / composite prognosis	CatBoost	validation set	0.737	APACHE II	0.75	-0.013	main article Fig. 2 CatBoost CCI AUC 0.737; supplementary Fig. 2 APACHE II CCI AUC 0.75; conventional score slightly higher
55	Zhang 2025	Mortality	CatBoost	validation set	0.804	APACHE II	0.78	0.024	main article Fig. 2 CatBoost mortality AUC 0.804; supplementary Fig. 2 APACHE II mortality AUC 0.78

Variable					Value				
57	Zhou 2024	28-day mortality SIC MIMIC-III	XGBoost	internal validation	0.828	SAPS II	0.746	0.082	best conventional comparator by AU-ROC was SAPS II
57	Zhou 2024	28-day mortality SIC MIMIC-IV	XGBoost	external validation	0.913	SOFA	0.773	0.14	best conventional comparator by AU-ROC was SOFA
57	Zhou 2024	28-day mortality SIC eICU-CRD	XGBoost	external validation	0.923	SAPS II	0.775	0.148	best conventional comparator by AU-ROC was SAPS II
69	Su 2021	ICU LOS >6 days	Random forest	single-center cohort	0.76	SOFA	0.62	0.14	full text: RF AUC 0.76; SOFA 0.62
69	Su 2021	Mortality	Random forest	single-center cohort	0.74	SOFA	0.7	0.04	full text: RF AUC 0.74; SOFA 0.70
69	Su 2021	Severity/septic shock	Random forest	single-center cohort	0.79	SOFA	0.59	0.2	full text: RF AUC 0.79; SOFA 0.59
83	Wang 2026	28-day mortality autoimmune sepsis external	Prediction model/nomogram	external validation	0.787	SOFA	0.679	0.108	full text: model AUC 0.787 vs SOFA 0.679
83	Wang 2026	28-day mortality autoimmune sepsis internal	Prediction model/nomogram	internal validation	0.771	SOFA	0.664	0.107	full text: model AUC 0.771 vs SOFA 0.664
83	Wang 2026	28-day mortality autoimmune sepsis training	Prediction model/nomogram	training	0.772	SOFA	0.664	0.108	full text: model AUC 0.772 vs SOFA 0.664
86	Yang 2023	28-day mortality SA-AKI	XGBoost	testing set	0.873	APS-III	0.713	0.16	full text: XGBoost AUC 0.873; APS-III 0.713; SAPS II 0.681
90	Sun 2025	7-day early mortality	ANN	training set	0.767	SOFA	0.718	0.049	best conventional comparator by AU-ROC was SOFA (APACHE II 0.710)
93	Hu 2022	In-hospital mortality	XGBoost	validation cohort	0.884	SOFA	0.77	0.114	full text: XGBoost 0.884; SOFA 0.770; SAPS-II 0.766; qSOFA 0.647

Abbreviations: AI, artificial intelligence; APACHE, Acute Physiology and Chronic Health Evaluation; APS, Acute Physiology Score; AUROC, area under the receiver operating characteristic curve; IQR, interquartile range; LODS, Logistic Organ Dysfunction System; ML, machine learning; OASIS, Oxford Acute Severity of Illness Score; qSOFA, quick Sequential Organ Failure Assessment; SAPS, Simplified Acute Physiology Score; SOFA, Sequential Organ Failure Assessment.

Footnote: ΔAUROC was calculated as AI/ML AUROC minus the best-performing conventional severity-score AUROC within the same study, outcome and validation cohort. The analysis is descriptive only because paired uncertainty estimates were generally unavailable.

Supplementary Table S8. PRISMA 2020 checklist

PRISMA checklist items are mapped to the manuscript sections.

Section and Topic	Item #	Checklist item	Location where item is reported
**TITLE**			
Title	1	Identify the report as a systematic review.	Title page, p. 1.
**ABSTRACT**			
Abstract	2	See the PRISMA 2020 for Abstracts checklist.	Abstract, pp. 1-2.
**INTRODUCTION**			
Rationale	3	Describe the rationale for the review in the context of existing knowledge.	Introduction, pp. 2-3.
Objectives	4	Provide an explicit statement of the objective(s) or question(s) the review addresses.	Introduction, p. 3 (final paragraph).
**METHODS**			
Eligibility criteria	5	Specify the inclusion and exclusion criteria for the review and how studies were grouped for the syntheses.	Methods, Sections 2.2, 2.4, 2.8 and 2.9, pp. 4-6.
Information sources	6	Specify all databases, registers, websites, organisations, reference lists and other sources searched or consulted to identify studies. Specify the date when each source was last searched or consulted.	Methods, Section 2.3, pp. 4-5; Supplementary Table S1.
Search strategy	7	Present the full search strategies for all databases, registers and websites, including any filters and limits used.	Supplementary Table S1.
Selection process	8	Specify the methods used to decide whether a study met the inclusion criteria of the review, including how many reviewers screened each record and each report retrieved, whether they worked independently, and if applicable, details of automation tools used in the process.	Methods, Section 2.5, p. 5; Figure 1, p. 9; Supplementary Table S2.
Data collection process	9	Specify the methods used to collect data from reports, including how many reviewers collected data from each report, whether they worked independently, any processes for obtaining or confirming data from study investigators, and if applicable, details of automation tools used in the process.	Methods, Section 2.6, p. 5.
Data items	10a	List and define all outcomes for which data were sought. Specify whether all results that were compatible with each outcome domain in each study were sought (e.g. for all measures, time points, analyses), and if not, the methods used to decide which results to collect.	Methods, Sections 2.7 and 2.8, p. 6; Supplementary Table S3.
	10b	List and define all other variables for which data were sought (e.g. participant and intervention characteristics, funding sources). Describe any assumptions made about any missing or unclear information.	Methods, Section 2.6, p. 5;

Section and Topic	Item #	Checklist item	Location where item is reported
			Supplemen-tary Table S3.
Study risk of bias assessment	11	Specify the methods used to assess risk of bias in the included studies, including details of the tool(s) used, how many reviewers assessed each study and whether they worked independently, and if applicable, details of automation tools used in the process.	Methods, Sec-tion 2.11, p. 7; Supplemen-tary Table S4.
Effect measures	12	Specify for each outcome the effect measure(s) (e.g. risk ratio, mean difference) used in the synthesis or presentation of results.	Methods, Sec-tions 2.7 and 2.10, pp. 6-7; Supplemen-tary Table S7.
Synthesis meth-ods	13a	Describe the processes used to decide which studies were eligible for each synthesis (e.g. tabulating the study intervention characteristics and comparing against the planned groups for each synthesis (item #5)).	Methods, Sec-tions 2.2, 2.4 and 2.8-2.10, pp. 4-7.
	13b	Describe any methods required to prepare the data for presentation or synthesis, such as handling of missing summary statistics, or data con-versions.	Methods, Sec-tions 2.6 and 2.7, pp. 5-6.
	13c	Describe any methods used to tabulate or visually display results of individual studies and syntheses.	Figure 1 and Tables 1-3 in the main man-uscript; Sup-plementary Tables S3-S7.
	13d	Describe any methods used to synthesize results and provide a rationale for the choice(s). If meta-analysis was performed, describe the model(s), method(s) to identify the presence and extent of statistical heterogeneity, and software package(s) used.	Methods, Sec-tion 2.13, pp. 7-8; Supple-mentary Table S6.
	13e	Describe any methods used to explore possible causes of heterogeneity among study results (e.g. subgroup analysis, meta-regression).	Methods, Sec-tions 2.8-2.10 and 2.13, pp. 6-8; Results, Sections 3.3 onward.
	13f	Describe any sensitivity analyses conducted to assess robustness of the synthesized results.	Not applica-ble: no formal sensitivity analysis was performed be-cause no meta-analysis

Section and Topic	Item #	Checklist item	Location where item is reported
			was undertaken (Methods, Section 2.13; Supplementary Table S6).
Reporting bias assessment	14	Describe any methods used to assess risk of bias due to missing results in a synthesis (arising from reporting biases).	Not formally assessed because the synthesis was descriptive and no meta-analysis was performed; this is acknowledged among review-process limitations in the Discussion.
Certainty assessment	15	Describe any methods used to assess certainty (or confidence) in the body of evidence for an outcome.	Methods, Section 2.14, p. 8: GRADE was not applied; translational readiness was evaluated narratively.
**RESULTS**			
Study selection	16a	Describe the results of the search and selection process, from the number of records identified in the search to the number of studies included in the review, ideally using a flow diagram.	Results, Section 3.1, pp. 8-9; Figure 1, p. 9.
	16b	Cite studies that might appear to meet the inclusion criteria, but which were excluded, and explain why they were excluded.	Results, Section 3.1, pp. 8-9; Supplementary Table S2.
Study characteristics	17	Cite each included study and present its characteristics.	Results, Section 3.3 onward; Table 1; Supplementary Table S3; References [19-93].

Section and Topic	Item #	Checklist item	Location where item is reported
Risk of bias in studies	18	Present assessments of risk of bias for each included study.	Results: risk-of-bias assessment; Supplementary Table S4.
Results of individual studies	19	For all outcomes, present, for each study: (a) summary statistics for each group (where appropriate) and (b) an effect estimate and its precision (e.g. confidence/credible interval), ideally using structured tables or plots.	Results: outcome and model performance sections; Supplementary Table S3.
Results of syntheses	20a	For each synthesis, briefly summarise the characteristics and risk of bias among contributing studies.	Results, Sections 3.2 onward; Tables 1-3; Supplementary Tables S3-S6.
	20b	Present results of all statistical syntheses conducted. If meta-analysis was done, present for each the summary estimate and its precision (e.g. confidence/credible interval) and measures of statistical heterogeneity. If comparing groups, describe the direction of the effect.	No meta-analysis performed; descriptive synthesis reported in Results and Supplementary Tables S3, S6 and S7.
	20c	Present results of all investigations of possible causes of heterogeneity among study results.	Results: outcome-, model-, validation- and translational-readiness syntheses; Supplementary Tables S3-S7.
	20d	Present results of all sensitivity analyses conducted to assess the robustness of the synthesized results.	Not applicable: no formal sensitivity analysis was performed.
Reporting biases	21	Present assessments of risk of bias due to missing results (arising from reporting biases) for each synthesis assessed.	Not formally assessed; acknowledged

Section and Topic	Item #	Checklist item	Location where item is reported
			as a limitation of the review process in the Discussion.
Certainty of evidence	22	Present assessments of certainty (or confidence) in the body of evidence for each outcome assessed.	Not formally graded; Methods, Section 2.14, p. 8 and Discussion.
**DISCUSSION**			
Discussion	23a	Provide a general interpretation of the results in the context of other evidence.	Discussion, interpretation of main findings.
	23b	Discuss any limitations of the evidence included in the review.	Discussion, Section 4.13, pp. 24-25.
	23c	Discuss any limitations of the review processes used.	Discussion, Section 4.13, pp. 24-25.
	23d	Discuss implications of the results for practice, policy, and future research.	Discussion and Conclusions, pp. 24-25.
**OTHER INFORMATION**			
Registration and protocol	24a	Provide registration information for the review, including register name and registration number, or state that the review was not registered.	Methods, Section 2.1, pp. 3-4: the review protocol was not prospectively registered.
	24b	Indicate where the review protocol can be accessed, or state that a protocol was not prepared.	No separate publicly accessible protocol is available; Methods, Section 2.1.
	24c	Describe and explain any amendments to information provided at registration or in the protocol.	Not applicable: no registered protocol was amended; the adult-only

Section and Topic	Item #	Checklist item	Location where item is reported
			refinement and additional Sco-pus/Cochrane searches are described in Methods and Figure 1.
Support	25	Describe sources of financial or non-financial support for the review, and the role of the funders or sponsors in the review.	Funding state-ment, p. 25.
Competing inter-ests	26	Declare any competing interests of review authors.	Conflicts of In-terest state-ment, p. 25.
Availability of data, code and other materials	27	Report which of the following are publicly available and where they can be found: template data collection forms; data extracted from included studies; data used for all analyses; analytic code; any other materials used in the review.	Data Availabil-ity Statement and Supple-mentary Mate-rials state-ment, p. 25; Supplemen-tary Tables S1-S8.

From: Page MJ, McKenzie JE, Bossuyt PM, Boutron I, Hoffmann TC, Mulrow CD, et al. The PRISMA 2020 statement: an updated guideline for reporting systematic reviews. BMJ 2021;372:n71. doi: 10.1136/bmj.n71. This work is licensed under CC BY 4.0. To view a copy of this license, visit https://creativecommons.org/licenses/by/4.0/
